# Pan-genome analysis of the sunflower *LOX* gene family: evolution, expression, and salt stress-responsive candidate genes

**DOI:** 10.3389/fpls.2026.1940299

**Published:** 2026-10-07

**Authors:** Min Song, Lifen He, Yanzun Wang, Wenjun Liu, Hongyuan Zheng, Wenhao Wang

**Affiliations:** Shanxi Key Laboratory of Oil Crops Genetic Improvement and Germplasm Enhancement, Institute of Industrial Crops, Shanxi Agricultural University, Taiyuan, China

**Keywords:** lipoxygenase, pan-genome, salt stress, selection pressure, sunflower

## Abstract

Sunflower (*Helianthus annuus* L.), the fourth largest edible oil-producing crop worldwide, has potential for application in the remediation of saline-alkali land. In this regard, lipoxygenase (LOX)-derived metabolites, particularly jasmonic acid, have been established to play positive roles in regulating the tolerance of plants to salt stress. However, using only a single reference genome provides limited genetic resolution, which is insufficient for a comprehensive elucidation of the evolutionary dynamics of the sunflower *LOX* family at the pan-genome level. In this study, we aimed to characterize the *HaLOX* gene family across multiple sunflower accessions and explore its evolutionary patterns in response to salt stress. Based on 10 sunflower genomes, we systematically identified the *HaLOX* family, revealing 220 genes clustered into 27 orthologous gene groups (OGGs) (12 core, 6 softcore, 7 shell, and 2 cloud OGGs). Together with the copy number variations analysis, these findings indicate that dispensable genes reflect the evolutionary diversity of the *LOX* family among sunflower accessions. Phylogenetic analysis further classified these OGGs into the 9-LOX and 13-LOX subfamilies (ratio, 1:2), a majority of which have undergone purifying selection. In contrast, *panHaLOX10* and *panHaLOX1*7 showed statistical support for episodic positive selection, suggesting possible functional divergence in specific sunflower lineages. Tandem duplication and whole-genome duplication (WGD)/segmental duplication were major drivers of *HaLOX* gene family expansion. Notably, only the 13-LOX subfamily underwent significant expansion via WGD/segmental duplication, whereas the 9-LOX subfamily did not experience such expansion events. In addition, transposon analysis suggested that DNA/hAT, LTR/Copia, and LTR/Gypsy may have contributed to *HaLOX* evolution, with proportions of 37.6%, 16.5%, and 16.9%, respectively. We also identified a high proportion of putative stress-responsive cis-regulatory elements (CREs) in *HaLOX* gene promoters (43.9%), among which MYB, MYC, and ABRE were the most abundant CREs. Moreover, RT-qPCR validated the expression patterns of nine 13-LOX genes exposed to salt stress, particularly *panHaLOX10/17*. Overall, our findings provide preliminary insights into the potential evolutionary implications of dispensable genes, thereby contributing to a better understanding of the evolutionary characteristics and functional differentiation of the *HaLOX* family, and potentially facilitating the identification of candidate genes for salt tolerance molecular breeding.

## Introduction

Lipoxygenases (LOXs; EC 1.13.11.12) are a highly conserved family of non-heme iron-containing dioxygenases that are widely distributed in animals, plants, and microorganisms. LOXs primarily catalyze the stereospecific oxidation of polyunsaturated fatty acids (PUFA) (Feussner and Wasternack, 2002). Plant LOXs possess a conserved N-terminal PLAT/LH2 domain and a C-terminal lipoxylase catalytic domain. The PLAT/LH2 domain is the non-catalytically essential structural domain of LOX proteins that mediates plasma membrane anchoring through reversible binding to lipid molecules or membrane surfaces. In contrast, the lipoxygenase domain comprises five highly conserved His residues, His-(X)_4_-His-(X)_4_-His-(X)_17_-His-(X)_8_-His, which catalyze dual oxygenation and constitute the essential core functional domain of LOX proteins (Andreou and Feussner, 2009). Linoleic acid (C18:2ω6) and α-linolenic acid (C18:3ω3) are the primary substrates for plant LOXs. These plant LOXs are classified into the following two major subfamilies based on their positional specificity for substrate oxygenation: 9-LOX and 13-LOX. The 9-LOX subfamily catalyzes the oxidation of PUFAs at the C9 position. This yields 9-hydroperoxyoctadecadienoic acid, 9-hydroperoxyoctadecatrienoic acid, and the downstream metabolite 9S-ketooctadecatrienoic acid. In contrast, 13-LOX acts at the C13 position to generate 13-hydroperoxyoctadecatrienoic acid, which is further converted into various signaling molecules, including jasmonates and green leaf volatiles (Wasternack and Feussner, 2018; Viswanath et al., 2020; Singh et al., 2022). Therefore, *LOX* family members primarily catalyze the oxygenation of PUFAs to produce various bioactive oxidized lipids. These lipids play crucial regulatory roles in plant growth and development, stress responses, and signaling molecule synthesis.

Athough the structural features and catalytic activities of LOX proteins are highly conserved across plants, distinct subfamilies exhibit pronounced differences in gene abundance and functional specificity. Six *LOX* genes have been identified in *Arabidopsis thaliana* (L.) Heynh (Bannenberg et al., 2009). Among these, *AtLOX1* and *AtLOX5* from the 9-LOX subfamily catalyze the production of oxylipins, particularly 9-hydroxyoctadecatrienoic acid, which plays a dual regulatory role in root development and defense responses (Vellosillo et al., 2007). *AtLOX1* is also involved in the early stages of stomatal closure during immune response (Montillet et al., 2013). The remaining four genes (*AtLOX2*, *AtLOX3*, *AtLOX4*, and *AtLOX6*) belong to the 13-LOX subfamily and function cooperatively in wound-induced jasmonic acid (JA) biosynthesis (Chauvin et al., 2013; Yang et al., 2020), with *AtLOX3* and *AtLOX4* specifically regulating male fertility via the JA pathway (Caldelari et al., 2011). In rice, 14 *LOX* genes that broadly participate in biotic and abiotic stress responses and seed longevity regulation have been identified (Umate, 2011). For example, *OsLOX9* enhances resistance to blast disease in rice via the JA biosynthesis pathway (Wang et al., 2025). Moreover, *OsLOX10* knockout in rice extended seed longevity, whereas its overexpression enhanced seedling tolerance to salinity stress (Wang et al., 2023a). Furthermore, *OsLOX1* gene knockout reduces seed viability and drought resistance, but delays seed viability and quality deterioration during storage (Mou et al., 2024; Weng et al., 2025). In pepper, *CaLOX1* enhances drought, salinity, and osmotic stress tolerance by abscisic acid (ABA) and stress-responsive genes, while reducing hydrogen peroxide accumulation and lipid peroxidation (Lim et al., 2015). In soybeans, *GmLOX6*, which encodes a chloroplast-localized 13-LOX protein, promotes JA synthesis to enhance antioxidant capacity, ion homeostasis, and membrane stability under salt stress (Li et al., 2025b). Similarly, the maize *ZmNAC032*-*ZmLOX3* module confers salt tolerance by activating JA signaling, enhancing reactive oxygen species scavenging, and maintaining Na^+^/K^+^ homeostasis (Che et al., 2025).

Studies on plant *LOX* gene families are largely confined to a single reference genome, thus failing to comprehensively reveal their genetic diversity at the pan-genome level. A single reference genome cannot adequately represent genetic variation at the population level. Hence, dispensable genes that are often associated with evolutionary adaptation among different accessions may be overlooked. Suitably, pan-genome-based family analyses effectively classify orthologous gene groups (OGGs) into core and dispensable categories and characterize gene presence/absence variants (PAVs) and copy number variations (CNVs) (Tong et al., 2025). With the construction and refinement of pan-genome maps for various species, the classification patterns and evolution of several important gene families, including barley basic helix-loop-helix (*bHLH*), *Brassica* trehalose-6-phosphate synthase (*TPS*), and *Fraxinus* ascorbate peroxidase (*APX*), were systematically elucidated at the pan-genome level (Tong et al., 2025; Hou et al., 2026; Xue et al., 2026).

The sunflower (*Helianthus annuus* L.) pan-genome, which was constructed from 10 representative accessions, revealed considerable genomic complexity and evolutionary adaptability (Huang et al., 2023). Sunflower have substantial value in the ecological restoration and improvement of saline-alkali soils, and the LOX-mediated JA biosynthesis pathway plays an important role in enhancing salt tolerance. Therefore, this study conducted a systematic whole-genome identification and characterization analysis of the *HaLOX* gene family at the pan-genome level. The study analyses included OGG identification and classification, CNVs and PAVs analysis, phylogenetic analysis, selection pressure analysis, gene duplication, and transposon distribution. Moreover, conserved protein domains and promoter cis-element features were characterized, tissue-specific and phytohormone-responsive expression profiles were analyzed, and salt stress-responsive *HaLOX* candidate genes were identified. This integrative approach aims to elucidate the evolutionary divergence and potential regulatory mechanisms of the *HaLOX* family at the pan-genome level. It may also facilitate the identification of candidate genes involved in salt stress responses. Ultimately, this provides a theoretical foundation and candidate genes for further functional studies of sunflower salt tolerance and advances molecular breeding for saline-alkali adaptation.

## Materials and methods

### Genome-wide identification of *HaLOX* genes

Genome assemblies and annotation datas of 10 sunflower accessions were retrieved from previously published studies (Badouin et al., 2017; Huang et al., 2023), with detailed genotype and version information provided in **Supplementary Table 1**. *HaLOX* genes were identified using two strategies: Hidden Markov Model (HMM) and BLASTP. First, the HMM profile of the conserved lipoxygenase domain (PF00305) was downloaded from the Pfam database (http://pfam.xfam.org/) (Paysan-Lafosse et al., 2025) to perform searches across 10 sunflower proteomes. Second, the *Arabidopsis* LOX (AtLOX) protein sequences of were downloaded from TAIR (https://www.arabidopsis.org/) (Umate, 2011), and used as queries to perform BLASTP searches (E-value ≤ 1e−10) against 10 sunflower proteomes (Camacho et al., 2009). After merging the putative HaLOX proteins obtained from both methods and removing redundant sequences, candidate sequences were submitted to the National Center for Biotechnology Information (NCBI) Conserved Domain Database (https://www.ncbi.nlm.nih.gov/cdd/), SMART Database (http://smart.embl.de/), and Pfam Database for domain integrity validation. Only sequences with a domain coverage of ≥80% were retained as candidate *HaLOX* genes for subsequent analyses. Additionally, the ProtParam tool in the ExPASy database (https://web.expasy.org/protparam/) was used to predict the theoretical isoelectric point and molecular weight of the candidate HaLOX proteins (Gasteiger et al., 2003).

### Orthologous gene groups identification and pan-genome classification

The OGGs of the sunflower *HaLOX* family were identified using OrthoFinder v3.1.0 (Emms and Kelly, 2015). Protein sequence alignment was performed using the MSA parameter, and all other parameters were set to their default values. A total of 220 *HaLOX* genes were identified across the 10 sunflower genomes and clustered into 27 orthologous gene groups (OGGs). Each OGG represents a set of orthologous *HaLOX* genes and may contain one or more copies from different accessions. Based on established classification criteria (Tong et al., 2025), the identified OGGs were categorized into four groups: core (present in all 10 accessions), softcore (present in nine accessions, ≥90%), shell (present in 2–8 accessions, >10% but <90%), and cloud (present in a single accession, ≤10%). The 27 OGGs were designated *panHaLOX1*–*panHaLOX27* according to a reproducible rule: OGGs were first grouped by pan-genome category (core, softcore, shell, and cloud, in that order), and within each category, OGGs were numbered sequentially in decreasing order of total copy number across the 10 genomes; when two or more OGGs had the same total copy number, they were ordered by their OrthoFinder cluster ID.

### Phylogeny, gene structure, protein domain, and conserved motif analyses

Gives the high level of sequence conservation within each OGG, we selected the longest protein sequences as a representative sequences for phylogenetic tree construction, gene structure analysis, protein domain identification, and conserved motif prediction. These representative genes are highlighted in red in **Supplementary Table 2**. In the event of multiple members of an OGG having identical sequence lengths, we preferentially selected the homologous sequence from the XRQv2 reference genome (Badouin et al., 2017; Huang et al., 2023). To provide a broader phylogenetic context and improve orthogroup-level resolution, we expanded the taxon sampling by including *Oryza sativa* subsp. *japonica* (rice) as a monocot representative, and *Tagetes erecta* (African marigold) and *Lactuca sativa* (lettuce) as additional Asteraceae representatives to increase within-Asteraceae coverage. These taxa were selected because they represent distinct phylogenetic positions relative to cultivated sunflower and have publicly available, well-annotated genome and protein resources. Protein sequences of *Oryza sativa* subsp. *japonica* were downloaded from the Ensembl Plant database (http://plants.ensembl.org/), those of *Tagetes erecta* were retrieved from the National Genomics Data Center (NGDC, https://ngdc.cncb.ac.cn/, accession number GWHDOCX00000000), and those of *Lactuca sativa* (V8 assembly) were obtained from the Phytozome database (https://phytozome-next.jgi.doe.gov). Complete LOX protein sequences were identified using a combined HMMER and BLASTP approach. To improve phylogenetic resolution, we extracted the lipoxygenase domains from all LOX proteins and aligned them using MAFFT (v7.526) with the -auto parameter (Katoh and Standley, 2013). A maximum likelihood phylogenetic tree was constructed using IQ-TREE (3.1.3) (Wong et al., 2026) with automatic model selection (-m MFP), -bb 1000 for ultrafast bootstrap, and 1000 SH-aLRT replicates (-alrt 1000); the best-fit substitution model selected by ModelFinder was Q.PLANT+I+R5. The tree was visualized and annotated using iTOL (https://itol.embl.de/).

Structural information on the *HaLOX* genes was retrieved from the Generic Feature Format version 3 (GFF3) files of each sunflower genome. All HaLOX protein sequences were submitted to the Pfam database to identify their characteristic domains, including the PLAT/LH2 and lipoxygenase domains (Paysan-Lafosse et al., 2025). Conserved motifs were predicted using the MEME Suite v5.4.1 (Bailey et al., 2015). The prediction parameters were set to -nmotifs 10 for representative OGG sequences and -nmotifs 6 for homologous proteins within individual OGGs; the remaining parameters were -mod anr -protein -minw 6 -maxw 200. The results of the gene structure, protein domain, and conserved motif analyses were visualized using TBtools v2.441 (Chen et al., 2020).

### Analysis of gene duplication and syntenic relationships

For each of the 10 sunflower pan-genome accessions, the longest transcript-encoded protein sequences of each gene were subjected to all vs.-all BLASTP searches (E-value ≤ 1e−10) (Camacho et al., 2009). Gene duplication events and their corresponding types were identified using MCScanX with the default parameters (Wang et al., 2012). Intra-genomic duplication patterns of *HaLOX* genes were visualized using Circos v0.69-8 (Krzywinski et al., 2009), and syntenic relationships across the pan-genome were characterized and visualized using the Python JCVI package v1.4.16 (Tang et al., 2024).

### *K*a/*K*s calculation

The coding sequences and corresponding protein sequences of the *HaLOX* family members were retrieved from the GFF3 of the 10 sunflower genomes. Bulk sequence alignment among *HaLOX* members within each OGG was performed using ParaAT v2.0 (Zhang et al., 2012), and *K*a/*K*s values were calculated using KaKs_Calculator v2.0 (model: YN) (Wang et al., 2010). The R packages ggridges and ggplot2 v4.0.3 were used to create ridgeline plots to visualize the distribution of *K*a/*K*s values (Valero-Mora, 2010), and the proportion of *HaLOX* genes with *K*a/*K*s values of >1 was displayed using a heatmap generated using ComplexHeatmap (v4.0.3) (Gu, 2022).

To rigorously assess the plausibility of positive selection, we performed likelihood-based codon substitution analyses using the HyPhy package (Pond et al., 2005) via the Datamonkey webserver (https://v3.datamonkey.org/). Three models were applied at three hierarchical levels—Branch-Site Unrestricted Statistical Test for Episodic Diversification (BUSTED) for detecting gene-wide positive selection, Adaptive Branch-Site Random Effects Likelihood (aBSREL) for detecting branch-specific positive selection, and Mixed Effects Model of Evolution (MEME) for detecting site-specific positive selection. All analyses were conducted using default parameters.

### Transposable elements identification

A sunflower-specific repeat library was generated via *de novo* prediction of the XRQv2 reference genome using RepeatModeler v2.0.2, with the -LTRStruct option (Flynn et al., 2020). This library was then used to annotate the transposable elements (TEs) in each sunflower genome assembly using RepeatMasker v4.1.2-p1 (Tarailo-Graovac and Chen, 2009), with RMBLAST as the search engine. For systematic TE profiling, TEs were surveyed within gene bodies and 2 kb flanking regions (upstream and downstream) of 219 *HaLOX* genes. Owing to its unanchored chromosomal location, *HanRHA438Chr00c04g0845171* was excluded from this analysis. TEs overlapping the target regions were extracted using BEDTools v2.30.0, and only TEs with confident classification annotations were retained for downstream analysis (Quinlan and Hall, 2010).

### Cis-regulatory elements analysis of the *HaLOX* gene family

For each sunflower genome, a 2 kb sequence upstream of the transcription start site of the *HaLOX* gene was extracted as the promoter sequence. These sequences were submitted to the PlantCARE database (https://bioinformatics.psb.ugent.be/webtools/plantcare/html/) (Lescot et al., 2002) for prediction of cis-regulatory elements (CREs). The identified elements were further classified into three major categories based on criteria described in previous studies (Mengarelli and Zanor, 2021). Subsequently, the number of different CREs was counted separately according to the sunflower genome, subfamily, and OGG classification.

### Expression patterns and co-expression network analysis

Transcriptome datasets obtained for sunflower (XRQ) tissues at different stages of development and phytohormone-treated roots were downloaded from the NCBI SRA database (SRP092742). RNA sequencing reads were mapped to the XRQv2 reference genome using HISAT2 v2.2.1 (Kim et al., 2015), and transcripts per million (TPM) values were calculated using the R package FeatureCounts (Liao et al., 2014). For the salt-stress analysis, transcriptome data from salt-stressed sunflower leaves were downloaded from the NCBI SRA database (SRP391119), and a salt stress-related TPM expression matrix was constructed using the same workflow. Expression heatmaps were generated using TBtools v2.441 (Chen et al., 2020). For weighted gene co-expression network analysis (WGCNA), the expression matrix was filtered to retain genes with standard deviation ≥ 3, and WGCNA was performed using the WGCNA R package (Langfelder and Horvath, 2008) to construct a gene co-expression network and identify co-expression modules. The soft-thresholding power was determined based on the scale-free topology criterion, defined as the lowest power at which the scale-free topology fit index (R²) initially reached 0.90. An unsigned network type was specified, and Pearson correlation was used for determining pairwise gene correlations. In addition, adjacency and topological overlap matrices (TOM) were calculated using the selected power, followed by average linkage hierarchical clustering. Modules were initially detected using the dynamic tree cut algorithm (deepSplit = 2, minimum module size = 30), and similar modules with module eigengene correlations > 0.85 were subsequently merged (cutHeight = 0.15). The *HaLOX* co-expression network (edge weight > 0.65) was visualized using Cytoscape v3.9.1 (Shannon et al., 2003).

### Plant cultivation, salt treatment, RNA isolation, and RT-qPCR analysis

Seedlings of the sunflower inbred line R30 were hydroponically grown to the four-leaf stage and exposed to 150 mM NaCl. Leaf samples were collected at 0, 0.5, 1, 2, 4, 8, 12, and 24 h after treatment and immediately frozen in liquid nitrogen and stored at –80 °C. Thereafter, total RNA was extracted from leaves using a TransZol Plant RNA Kit (TransGen Biotech, Cat. No. ET121), and the first-strand complementary DNA (cDNA) was synthesized using TransScript First-Strand cDNA Synthesis SuperMix (TransGen Biotech, Cat. No. AE341), according to the manufacturer’s protocol. RT-qPCR was performed using TransScript^®^ Green One-Step qRT-PCR SuperMix Kit (TransGen Biotech, Cat. No. AQ211), and the *HaTubulin* gene was used as an internal reference for normalization. For each salt treatment time point, three independent biological replicates were collected. Each replicate consisted of an independent sample of leaves pooled from five R30 seedlings. The seedlings for each replicate were obtained from different hydroponic batches and subjected to independent salt treatments. The relative expression level was calculated using the 2^–ΔΔCt^ method (Livak and Schmittgen, 2001). All primer sequences are listed in **Supplementary Table 14**.

## Results

### Identification and characterization of the *HaLOX* gene family in the sunflower pan-genome

A total of 220 HaLOX genes were identified across 10 sunflower genome assemblies, with 19–24 copies per genome. Among these, the OQP8 genome contained the fewest HaLOX genes (19), and the HA300 genome contained the most genes (24) (**Figure 1A**). Chromosomal localization analysis revealed that HaLOX genes were distributed across nine chromosomes, with the highest number on Chr11 and Chr13 (**Supplementary Table 3**). Physicochemical analysis showed that the HaLOX proteins consisted of 593–938 amino acids, with molecular weight and isoelectric point of 67.67–105.73 kDa and 4.61–7.90, respectively (**Supplementary Table 3**).

**Figure 1:**
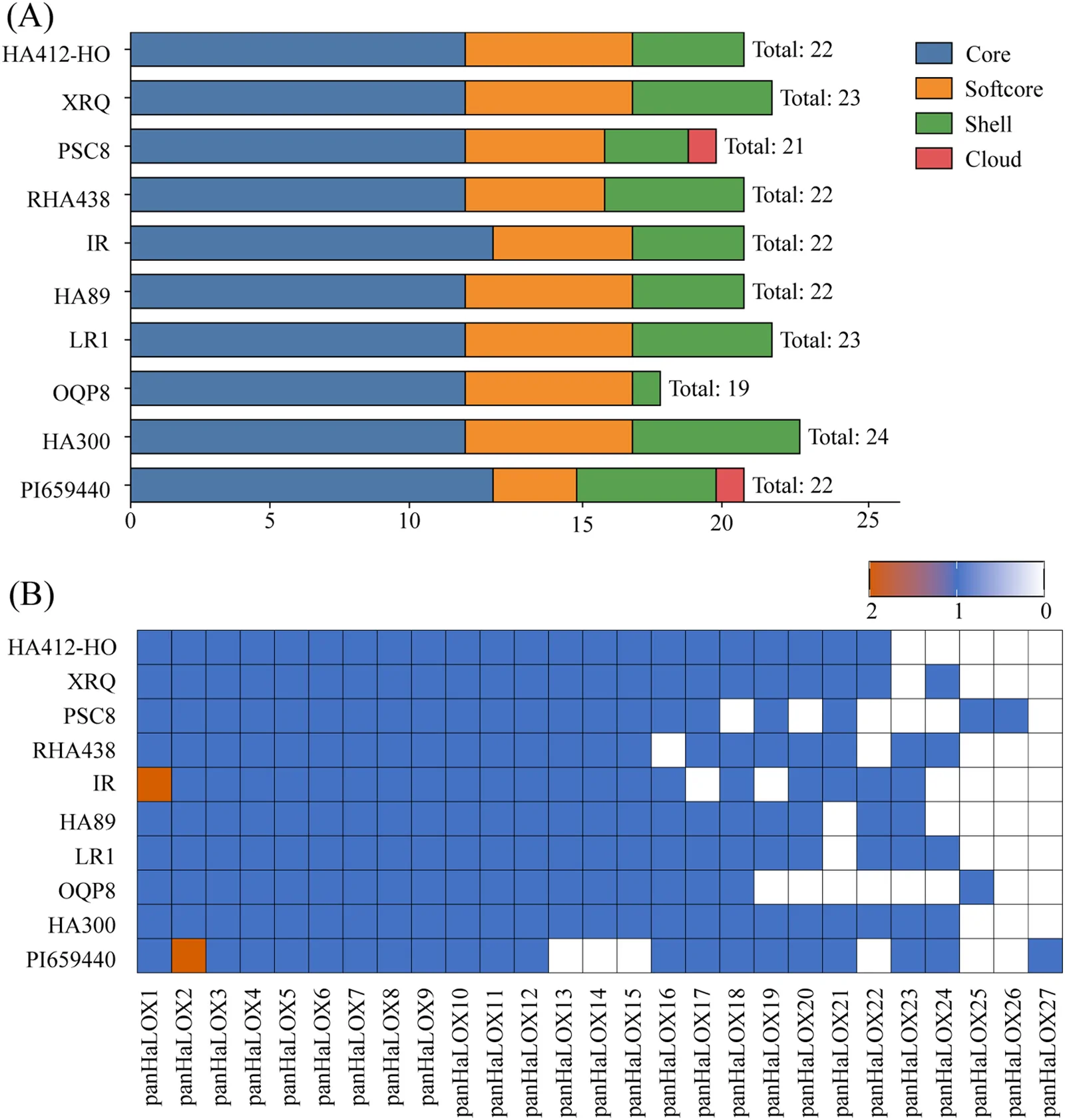
Classification and copy number variation (CNV) of *HaLOX* genes in the sunflower pan-genome. **(A)** Number of core, softcore, shell, and cloud genes across each genome. **(B)** Heatmap showing CNVs of each *HaLOX* orthologous gene group (OGG) across the 10 sunflower genomes.

The 220 HaLOX genes were classified into 27 OGGs (named panHaLOX1–27), a number that exceeded the number of HaLOX genes found in any individual genome (**Figure 1B**). Based on the pan-genome classification criteria, the 27 OGGs were categorized into 12 core (panHaLOX1–12), 6 softcore (panHaLOX13–18), 7 shell (panHaLOX19–25), and 2 cloud (panHaLOX26–27) genes (**Supplementary Table 2**). The core genes were highly conserved across the genomes, with only panHaLOX1 and panHaLOX2 showing copy number expansion (two copies each) in IR and PI659440, respectively. Each genome contained 5–6 softcore genes, except for PI659440, which contained three softcore genes. In contrast, the number of shell genes per genome showed the greatest variation (1–6), with OQP8 containing one and HA300 containing six. Cloud genes were present only in PSC8 and PI659440. Based on CNV and PAV analyses, the copy number of each OGG within an individual genome ranged from 0 to 2 (**Figure 1B**). These results indicate that the HaLOX gene family exhibits relatively abundant CNVs and PAVs within sunflower species, thus reflecting its functional diversity.

### Pan-genome-based phylogenetic classification of the *HaLOX* gene family

To elucidate the evolutionary relationships among HaLOX genes, we reconstructed a maximum likelihood phylogenetic tree using 83 LOX protein sequences. Besides the 27 reference HaLOX sequences from sunflower, 6, 12, 17, and 21 sequences were retrieved from Arabidopsis, rice, marigold, and lettuce, respectively (**Supplementary Table 4**). Evolutionary analysis revealed that all OGGs could be classified into the two subfamilies, 9-LOX and 13-LOX (Type I and Type II) (**Figure 2A**; **Supplementary Figure 1**). Among the 27 representative OGGs, the 9-LOX subfamily contained only 9 OGGs (33.33% of the total), whereas 13-LOX subfamily Type I and Type II, contained 15 (55.56%) and 3 (11.11%) OGGs, respectively, accounting for approximately two-thirds of the total. This indicated that compared with the 9-LOX proteins, the latter proteins may play a more prominent role in sunflower biology. Within the 9-LOX subfamily, panHaLOX11 (core) and LsLOX16 formed an isolated branch, potentially representing lineages specific to certain Asteraceae crops. We also identified three closely related shell OGGs (panHaLOX20/23/25), which may provide evidence of duplication events in sunflower. The remaining four core OGGs (panHaLOX2/7/8/9) and one softcore OGG (panHaLOX18) were found to be closely related to corresponding LOX proteins in lettuce and marigold. Within the 13-LOX subfamily, panHaLOX3 (core), panHaLOX13/14 (softcore), panHaLOX19 (shell), and panHaLOX27 (cloud) clustered within a single subclade, as did panHaLOX22/24 (shell) and panHaLOX26. Similarly, LsLOX10/11/14/15 in lettuce clustered together, possibly indicating local duplication within the 13-LOX subfamily Type I. The remaining 13-LOX genes showed close evolutionary relationships with other LOX proteins in plants within the family Asteraceae. In terms of pan-gene family classification, whereas core, softcore, and shell OGGs were distributed among all subfamilies, cloud OGGs were detected exclusively among 13-LOX Type I genes, highlighting the contribution of Type I to lineage-specific OGGs. Furthermore, each of the 10 sunflower genomes contained 6–9 genes in the 9-LOX subfamily and 12–16 genes in the 13-LOX subfamily (**Figure 2B**), thus reflecting the expansions and contractions of gene copy numbers of HaLOX subfamilies among sunflower accessions.

**Figure 2:**
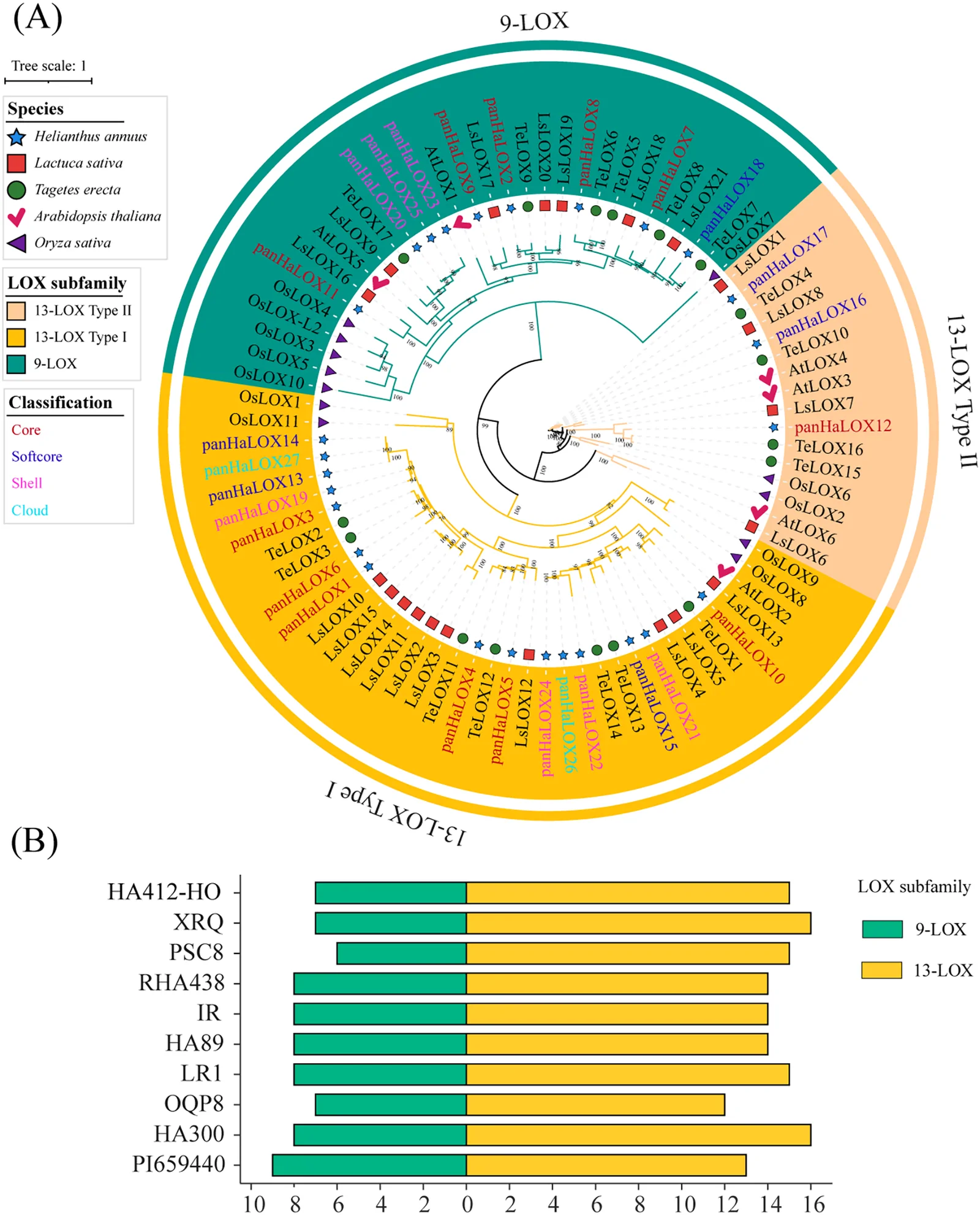
Phylogenetic relationships and subfamily classification of *HaLOX* genes across the sunflower pan-genome. **(A)** Phylogenetic tree of LOX proteins from rice, marigold, lettuce, *Arabidopsis*, and sunflower. **(B)** Number of 9-LOX and 13-LOX genes across different sunflower genomes.

### Conserved motifs, protein domains, and gene structure analyses of the *HaLOX* gene family

Analysis of the conserved motifs and protein domains across the representative sequences of 27 OGGs revealed that all members, except panHaLOX26, which lacked motifs 8 and 9 and the PLAT/LH2 domain, contained 10 conserved motifs and the PLAT/LH2 and lipoxygenase domains (**Figure 3B**; **Supplementary Figure 2**). This indicated that motifs 8 and 9 were associated with the PLAT/LH2 domain. Additionally, panHaLOX2/7/8/9/18/20/23/25 contained an extra motif 7 at their C-terminus, whereas panHaLOX17 carried a motif 3 at its C-terminus (**Figure 3A**). Gene structure analysis revealed that variations in the lengths of introns 1 and 2 were the primary determinants of length differences among panHaLOX genes. Specifically, panHaLOX2, panHaLOX4, and panHaLOX24 were the longest genes owing to the great length of these two introns. In general, panHaLOX9, panHaLOX12, and panHaLOX16 possessed the shortest gene lengths (**Figure 3C**).

**Figure 3:**
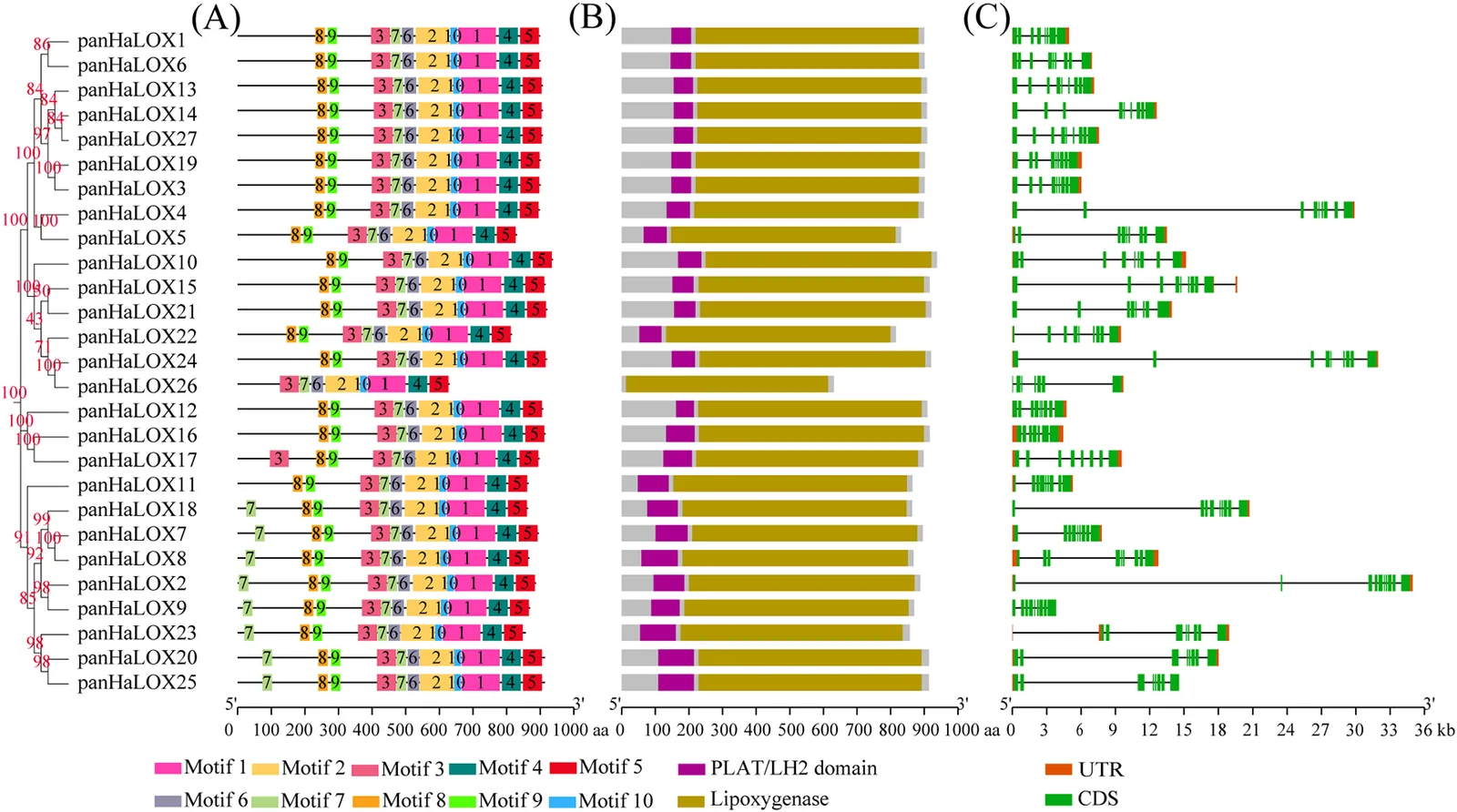
Conserved motifs, protein domains, and gene structures of the 27 sunflower *HaLOX* orthologous gene groups (OGGs). **(A)** Distribution of 10 conserved motifs. **(B)** Schematic of conserved domains. **(C)** Structure of *HaLOX* genes. Green boxes indicate exons, black lines represent introns, and orange boxes denote untranslated regions (UTR). A neighbor-joining (NJ) phylogenetic tree was generated from 27 OGG proteins.

To elucidate the genetic diversity of HaLOX among different sunflower accessions, the sequence structural variations of 27 OGGs were systematically compared across 10 sunflower genomes. The results demonstrated pronounced differences between panHaLOX12, panHaLOX13, and panHaLOX16 across different genomes. Notably, panHaLOX12 lacks motif 4 and the PLAT/LH2 domain in LR1, OQP8, and HA300, resulting from the loss of two exons at the 5′ end of the genes. whereas members in the remaining seven genomes are highly conserved (**Supplementary Figure 3**). PanHaLOX13 was absent in PI659440, whereas its N-terminal region lacked motif 6 in HA89, LR1, OQP8, and HA300. This variation may result from sequence differences in the first exon; however, the variation does not affect the presence of the two key domains of the LOX protein. In contrast, the motifs, domains, and gene structures of panHaLOX13 were highly conserved across the remaining five accessions (**Supplementary Figure 4**). Similarly, panHaLOX16 was absent in RHA438. However, in HA89, LR1, and HA300, sequence differences in the first exon of panHaLOX16 result in the loss of one motif 6 at the N-terminal region of its protein, but this variation does not affect the presence of the two key domains (**Supplementary Figure 5**).

### Gene duplication and syntenic relationships of the *HaLOX* gene family

To elucidate the expansion mechanism of the HaLOX gene family across the pan-genome, duplication analysis of 220 HaLOX genes was performed. This revealed that tandem and whole-genome (WGD)/segmental duplications were the predominant expansion modes in this family and accounted for 35.0% and 34.5%, respectively. This was followed by dispersed (16.4%) and proximal (14.1%) duplications (**Figure 4A**; **Supplementary Table 3**). Further analysis revealed substantial differences in duplication modes across HaLOX gene family categories (**Figure 4B**). In particular, core genes predominantly originated from tandem (32.0%) and dispersed duplications (27.9%), followed by WGD/segmental (24.6%) and proximal (15.6%) duplications. Softcore genes were primarily derived from WGD/segmental duplications (50.0%), with tandem (35.2%) and proximal (14.8%) duplications accounting for the remaining proportion, whereas no dispersed duplication events were detected. Shell genes exhibited tandem (45.2%) and WGD/segmental (40.5%) duplications and collectively represented over 85% of the duplication events. Cloud genes are exclusively derived from WGD/segmental events. At the subfamily level, 76 9-LOX genes primarily originated from tandem (50.0%) and dispersed (42.1%) duplications, with the remaining six genes arising from proximal duplication. Notably, no WGD/segmental duplication events were detected in this subfamily. In contrast, among the 144 13-LOX genes, WGD/segmental duplication accounted for the highest proportion (52.8%), followed by tandem duplication (27.1%) and proximal (17.4%) duplication, whereas only four genes (2.8%) resulted from dispersed duplication (**Figure 4C**). In summary, WGD/segmental duplication served as the predominant and distinguishing expansion mechanism for the 13-LOX subfamily, whereas the 9-LOX subfamily expanded mainly through tandem and dispersed duplications without WGD/segmental events.

**Figure 4:**
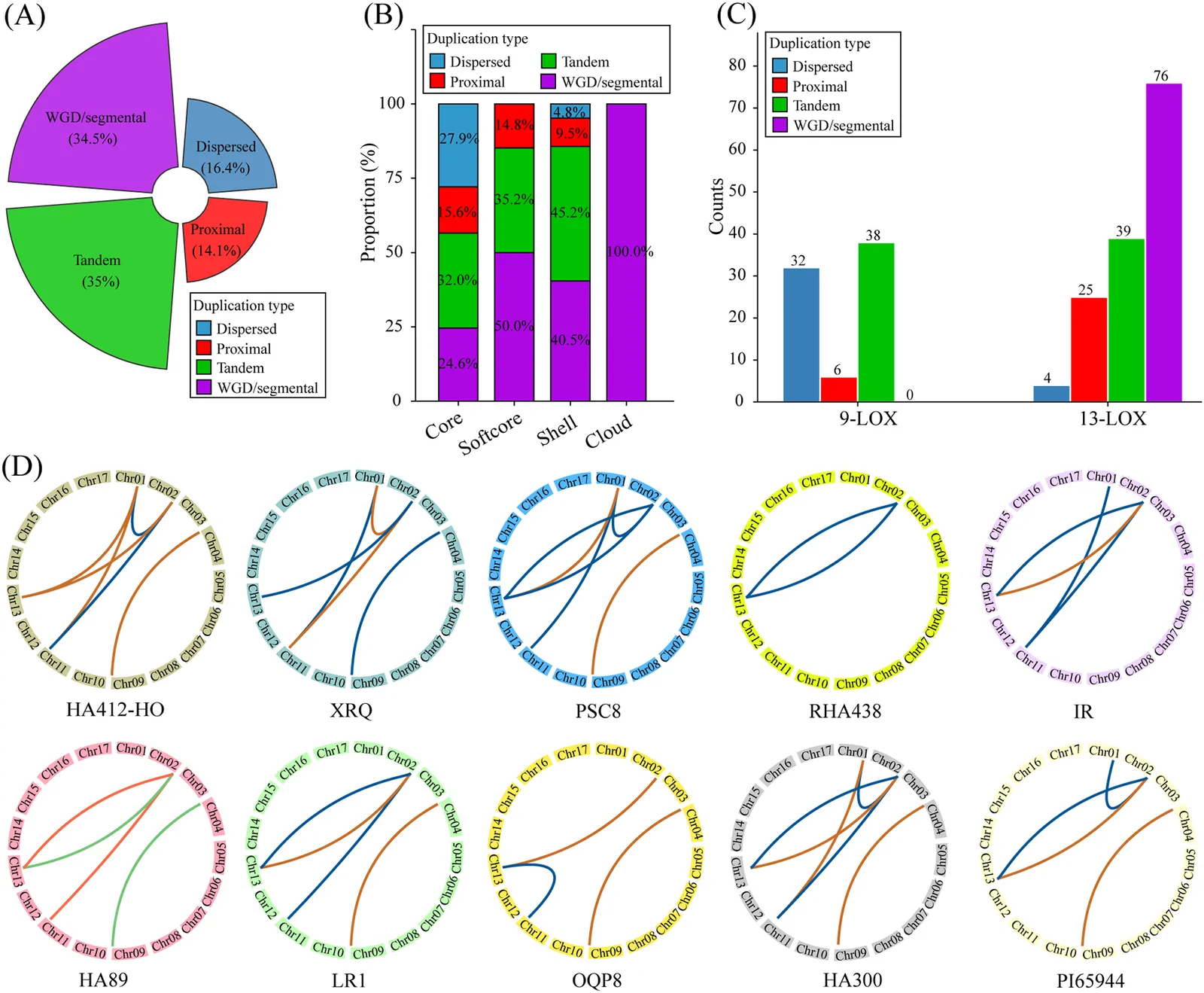
Characterization of gene duplication and syntenic relationships of the *HaLOX* pan-genes. **(A)** Four gene duplication types identified in sunflower pan-genome. **(B)** Proportional distribution of duplication types among core, softcore, shell, and cloud genes. **(C)** Counts of duplication events for the two subfamilies. **(D)** Circos plots showing syntenic relationships of *HaLOX* genes across 10 genomes. Each circle represents a genome. Brown and blue lines indicate paralogous gene pairs with identical and distinct pan-gene classifications, respectively.

Furthermore, Circos visualization revealed that syntenic relationships predominantly occurred on six different chromosomes (**Figure 4D**; **Supplementary Table 5**). This suggests that these chromosomes may be the primary regions involved in HaLOX gene expansion. Based on the synteny analysis of the 10 sunflower genomes, 12 syntenic relationships were identified. These included three syntenic loci containing a single orthologous gene per genome on Chr03, Chr10, and Chr16, and three syntenic gene clusters containing three tandemly arranged orthologous genes per genome on Chr02, Chr11, and Chr13 (**Supplementary Figure 6**; **Supplementary Table 6**). Among these, 11 syntenic relationships belonged to core genes, with only one occurring between the core and shell genes. This further indicated the conservation of core genes across the pan-genome.

### Transposable elements analysis of the *HaLOX* gene family

TEs accounted for more than 75% of the sunflower genome (Badouin et al., 2017). In this study, 1,645 classified TEs were identified within or adjacent to HaLOX genes, with each HaLOX gene containing 1–22 TEs distributed within or near the gene (**Supplementary Table 7**; **Figure 5A**). Class II DNA transposons accounted for 56.8% (934 elements) and primarily comprised DNA/hAT (37.6%), DNA/CMC-EnSpm (6.0%), DNA/MULE-MuDR (5.3%), DNA/PIF-Harbinger (4.3%), DNA/Maverick (1.9%), RC/Helitron (1.0%), and DNA/others (0.6%). Class I retrotransposons comprised 43.2% (711 elements), including LTR/Gypsy (16.9%), LTR/Copia (16.5%), LINE/RTE (6.4%), LINE/L1 (2.2%), LINE/CRE (0.6%), and LTR/others (0.5%). Considering the distinct distribution patterns of Helitron and LINE elements in the HaLOX family, TEs were further classified into four categories: DNA transposons (excluding Helitrons), Helitrons, LTR retrotransposons, and LINE; their distribution across different duplication types and subfamilies was then compared.

**Figure 5:**
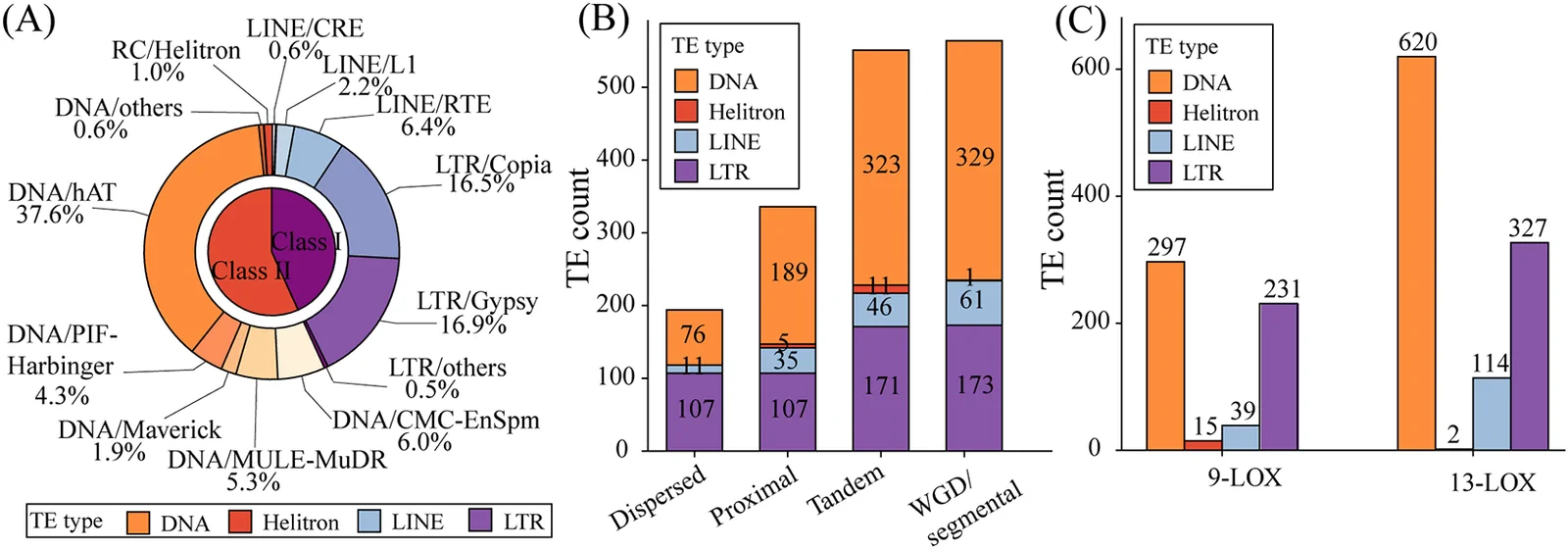
Transposable element (TE) analysis of *HaLOX* genes in the sunflower pan-genome. **(A)** Classification and relative abundance of TE types within 2 kb flanking regions of *HaLOX* loci in the sunflower pan-genome. **(B)** Distribution of TE types among different duplication modes. **(C)** Counts of TE types in the 9-LOX and 13-LOX subfamilies.

Tandem and WGD/segmental duplications were the two major duplication types in the HaLOX gene family. Therefore, HaLOX genes associated with these two types harbored the highest number of TEs within their gene and flanking regions. In contrast, relatively fewer TEs were associated with genes derived from proximal or dispersed duplications (**Figure 5B**). At the subfamily level, the 9-LOX and 13-LOX subfamilies contained 582 and 1,063 TEs, respectively, with TE-to-gene ratios of 7.66 and 7.38, respectively, which were highly comparable. These results suggest that TE accumulation in different subfamilies primarily scales with gene copy number expansion (**Figure 5C**). Among the four TE types, DNA transposons (excluding Helitrons) and LTR retrotransposons were the two most abundant categories in both the 9-LOX and 13-LOX subfamilies, followed by LINE. In contrast, Helitron transposons exhibited the opposite distribution pattern, with 15 in the 9-LOX subfamily and only two in the 13-LOX subfamily (**Figure 5D**). This suggests that Helitron transposons may have played a role in the evolution of certain 9-LOX genes.

### Analysis of selection pressures acting on *HaLOX* genes

The Ka/Ks ratio is a key indicator of the evolutionary constraints of a gene. Based on 10 sunflower genomes, the Ka/Ks values of 25 OGGs were calculated (**Supplementary Table 8**). The panHaLOX26 and panHaLOX27 were excluded owing to insufficient homologous sequence pairs. The Ka/Ks values ranged from 0 to 4.5, with the majority concentrated below 1, and pronounced peak differences were observed across different OGGs (**Figure 6A**). Notably, panHaLOX10, panHaLOX14, and panHaLOX17 had Ka/Ks ratios >1, suggesting that these genes may have undergone positive selection or adaptive functional divergence. Among these, panHaLOX10 showed a particularly high frequency of Ka/Ks ratios >1 in HA300 (**Figure 6B**), implying a lineage-specific selection pressure on this gene. In contrast, the remaining 22 genes had Ka/Ks values <1 across all accessions, indicating a strong purifying selection that likely maintained their conserved protein functions. These findings provide evolutionary evidence for the identification of candidate HaLOX genes involved in sunflower domestication and agronomic trait improvement.

**Figure 6:**
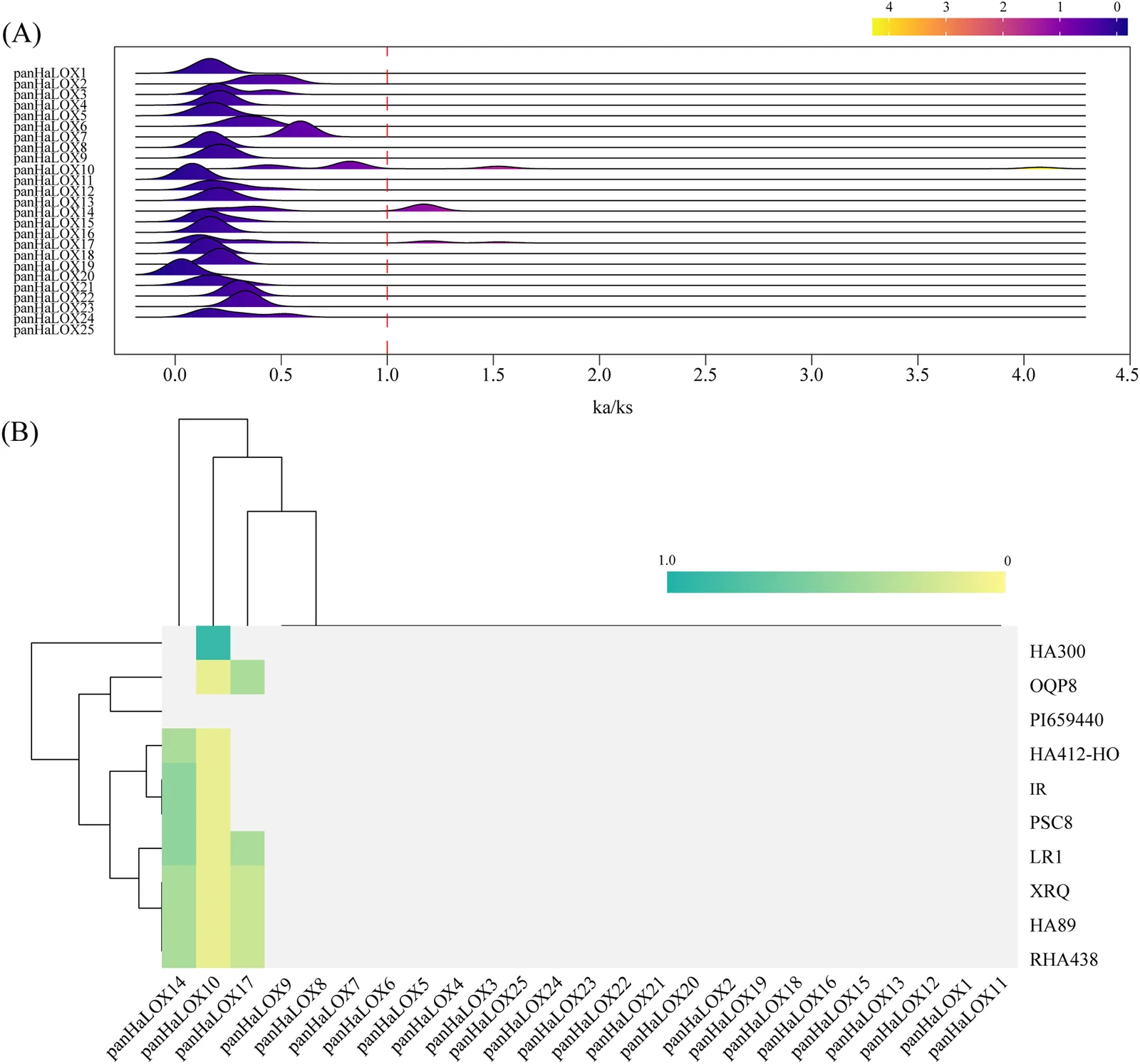
*K*a/*K*s analysis of *HaLOX* genes in sunflower. **(A)** Distribution of *K*a/*K*s ratios across 10 sunflower accessions. The x-axis shows the *K*a/*K*s ranges, and the height and color intensity of the peaks indicate the number of gene pairs in different sunflower accessions. **(B)** Heatmap of the proportion of *HaLOX* genes with *K*a/*K*s *>*1 for each orthologous gene group (OGG) across the 10 sunflower accessions. The x-axis shows OGGs, and the y-axis represents accessions. The colored blocks indicate the proportion of orthologous gene pairs with *K*a/*K*s *>*1.

To further examine the evidence for positive selection suggested by Ka/Ks ratios, we performed likelihood-based codon substitution analyses using the HyPhy package at three levels—gene-wide, branch-specific, and site-specific. BUSTED analysis provided evidence of significant episodic positive selection in panHaLOX10 (LRT = 29.84, p < 0.001) and panHaLOX17 (LRT = 38.44, p < 0.0001), whereas no significant signal was detected for panHaLOX14 (p = 0.5) (**Supplementary Table 9**). For panHaLOX10, 5.5% of codon sites were inferred to be under strong positive selection (ω = 489.07, p < 0.001), whereas for panHaLOX17, 0.6% of sites showed evidence of strong positive selection (ω = 2632.46, p < 0.0001), with the remaining sites primarily inferred to be under purifying selection (**Supplementary Table 9**). aBSREL analysis indicated that the signal of episodic positive selection in panHaLOX10 was restricted to a single ancestral branch (Node6, corresponding to the most recent common ancestor of HA300, OQP8, HA89, and LR1, LRT = 54.14, p < 0.0001), whereas in panHaLOX17, evidence for episodic positive selection was detected on three branches (HA300, RHA438, and Node9, which corresponds to the common ancestor of LR1 and OQP8), including two terminal branches, suggesting lineage-specific selection evolution (**Supplementary Table 9**). MEME selection analysis further identified a single significant positively selected site in panHaLOX10 (site 111; Thr→Leu, p = 0.0252) and two significant sites in panHaLOX17 (site 20; Ser→His, p = 0.0041; site 899; Val→Gln, p = 0.0004). Moreover, these sites had estimated synonymous substitution rates of zero (α = 0.00) and markedly high non-synonymous substitution rates (high β^+^ values), further supporting evidence for episodic positive selection at these codon positions (**Supplementary Table 9**). Collectively, these analyses provide evidence for episodic positive selection in panHaLOX10 and panHaLOX17, whereas no significant evidence for positive selection was detected for panHaLOX14 under the likelihood-based framework. These results should therefore be interpreted as molecular signatures of episodic selection rather than as direct evidence for adaptive phenotypic effects of specific substitutions.

### Analysis of cis-regulatory elements in *HaLOX* gene promoters

To understand the transcriptional regulatory mechanisms of the HaLOX gene family, prediction of CREs was performed on the promoter regions of 220 HaLOX genes, and 9,830 putative CREs spanning 40 distinct but well-defined elements were identified (**Supplementary Table 10**). Based on functional annotation, these CREs were classified into three major categories: abiotic and biotic stress responses (4,313, accounting for 43.9%), phytohormone-responsiveness (2,634, accounting for 26.8%), and responses to plant growth and development (2,883, accounting for 29.3%) (**Figure 7A**). Analysis of the number of elements across categories revealed that MYB (1,531), MYC (915), and ARE (608) ranked highest in the stress response category, whereas the top elements in the phytohormone-responsive category were ABRE (762), CGTCA-motif (400), TGACG-motif (400), and as-1 (400). In the growth and development category, the top three elements were G-box (905), Box 4 (503), and GT1-motif (368) (**Supplementary Figure 7**). Furthermore, statistical analysis across the 10 sunflower genomes revealed that the relative proportions of the three CRE categories remained largely conserved across accessions (**Figure 7B**). However, their absolute numbers exhibited accession-specific variation: PSC8 and OQP8 had the lowest total number, whereas HA300 exhibited a substantially higher total count than those of the other accessions. The number of genes in the 13-LOX subfamily (144) was approximately 1.9 times that in the 9-LOX subfamily (76). When compared on a per-gene basis, the 13-LOX subfamily contained approximately 1.9-fold more MYC elements per gene (719 and 196 in total, respectively) than the 9-LOX subfamily (**Figure 7C**; **Supplementary Table 10**). Considering that MYC is the binding site for core transcription factors in the JA signaling pathway (Zhou et al., 2024), its pronounced abundance in the promoters of 13-LOX genes may suggest that members of this subfamily are potentially under JA-dependent transcriptional regulation in sunflower, although this inference awaits functional validation. Analysis of the top 10 most-abundant CREs revealed that the core gene promoter region exhibited the highest total number of CREs, with a particularly notable enrichment of stress-responsive MYB elements. In particular, the number of MYB elements in core genes, such as panHaLOX1, panHaLOX8, and panHaLOX11, exceeded 100. Softcore and shell genes exhibited a moderate number of CREs, whereas cloud genes showed markedly fewer CREs across all categories than those of other types. Furthermore, growth-and development-related G-box elements and phytohormone response-related ABRE elements were specifically enriched in the softcore gene panHaLOX18 and the shell gene panHaLOX19, respectively (**Figure 7D**). Overall, differences in the composition of CREs among different categories of the HaLOX gene family were observed, which may be associated with potential differences in gene expression under environmental conditions. However, promoter element prediction alone does not demonstrate actual transcription factor binding or pathway activation. Therefore, these inferences require further experimental validation.

**Figure 7:**
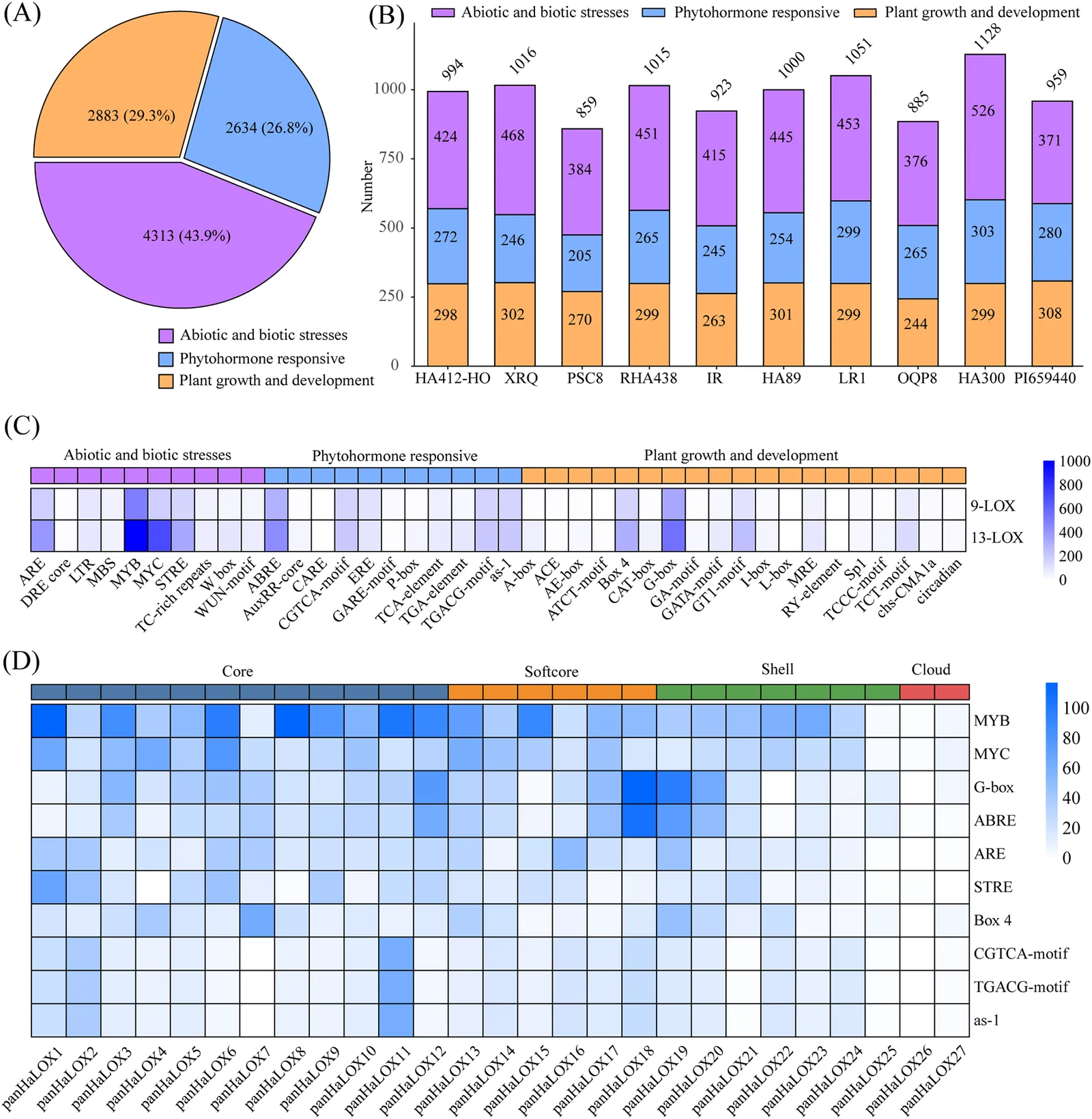
Distribution of cis-elements in *HaLOX* gene promoters across the sunflower pan-genome. **(A)** Proportional distribution of three major functional categories of cis-elements: abiotic and biotic stresses (purple), phytohormone-responsive (blue), and plant growth and development (orange). **(B)** Number of cis-elements in each of the three categories across the ten sunflower genomes. **(C)** Cis-element abundance across 9-LOX and 13-LOX subfamilies, with color intensity representing element counts. **(D)** Distribution of the top 10 most-abundant cis-elements among 27 orthologous gene groups (OGGs), with color intensity representing element counts.

### Tissue-specific and phytohormone-responsive expression patterns of *HaLOX* genes

The sunflower reference genome XRQv2 lacks panHaLOX23, panHaLOX25, panHaLOX26, and panHaLOX27; consequently,no expression data were available for these genes in this accession. The remaining HaLOX genes exhibited tissue-specific expression patterns (**Figure 8A**; **Supplementary Table 11**). Specifically, panHaLOX6/8/18/22 showed root-specific expression, panHaLOX4 showed leaf-specific expression, panHaLOX7 showed specific expression in the roots and pistil, panHaLOX9 showed specific expression in the pollen and stamen, panHaLOX21 showed specific expression in the roots and stamen, and panHaLOX24 was substantially expressed in the pistil and ligule. Notably, panHaLOX16 and panHaLOX17 shared similar tissue expression patterns and also exhibited the closest phylogenetic relationships. However, despite their similar expression patterns, the two genes differed in the predicted numbers of cis-regulatory elements (**Figure 7D**). Collectively, these expression patterns suggest functional specialization of HaLOX genes across sunflower tissues.

**Figure 8:**
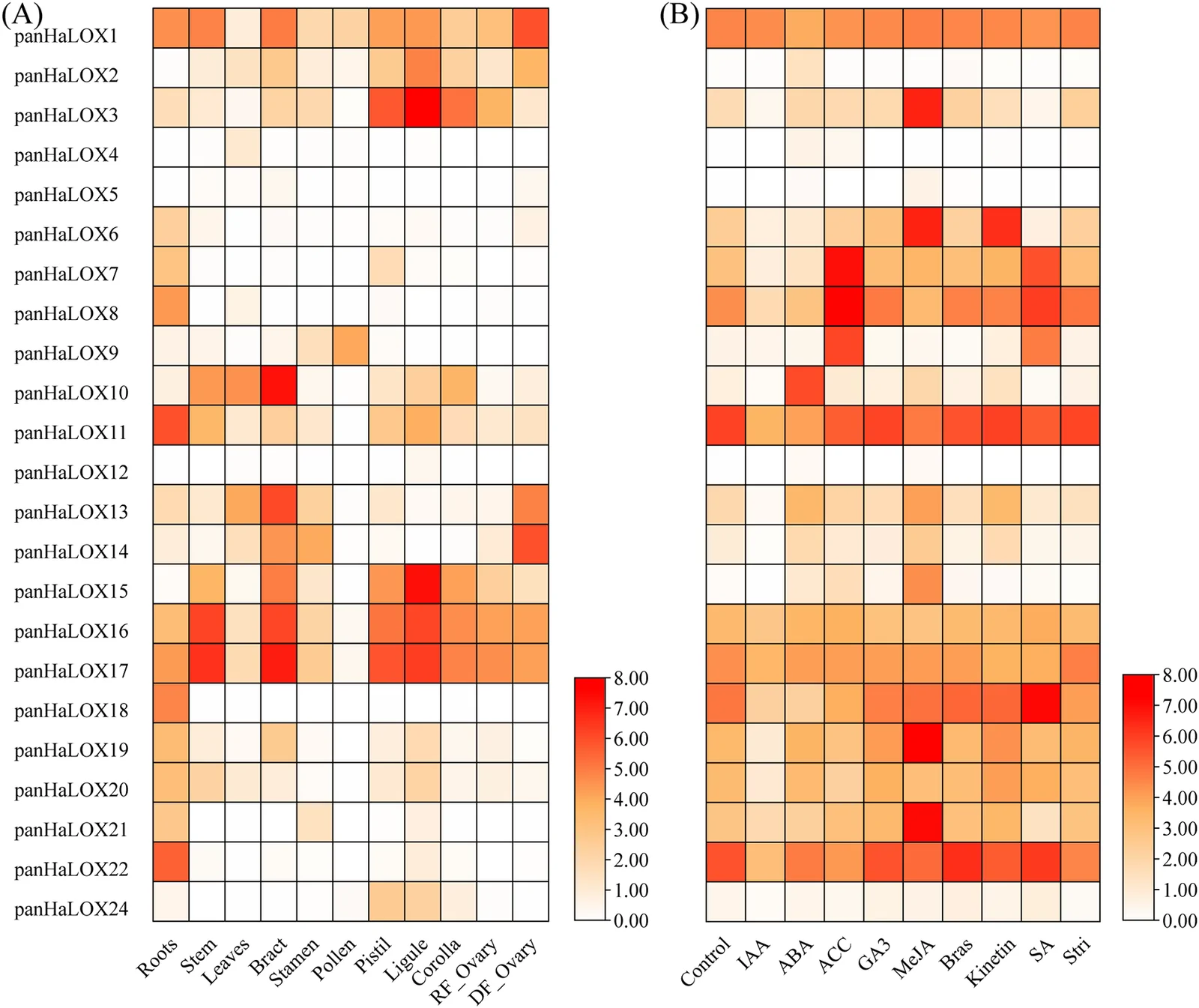
Expression patterns of *HaLOX* genes in different tissues and under phytohormone treatments. **(A)** Tissue-specific expression patterns of *HaLOX* genes across 11 sunflower tissues. Ray floret ovary (RF_Ovary) and Disc floret ovary (DF_Ovary) corresponds with developing seeds. **(B)** Expression profiles of *HaLOX* genes in response to nine phytohormone treatments in roots, including auxin (IAA), abscisic acid (ABA), ethylene (ACC), gibberellin acid (GA3), methyl-jasmonate (MeJA), brassinosteroid (Bras), kinetin, salicylic acid (SA), and strigolactone (stri), with untreated control as reference. Gene expression values were normalized using log_2_(TPM + 1) transformation. The color intensity indicates expression levels ranging from low (white) to high (red).

Considering the enrichment of multiple phytohormone-responsive elements in HaLOX promoters, the expression patterns of sunflower roots in response to nine exogenous phytohormone treatments were further analyzed (**Figure 8B**, **Supplementary Table 12**). Following auxin (IAA) treatment, the expression of most HaLOX genes was downregulated or showed no notable changes. ABA treatment induced the most pronounced upregulation of panHaLOX10. Kinetin treatment induced the highest upregulation of panHaLOX6. Following treatment with ethylene (ACC) or salicylic acid (SA), expression of panHaLOX7/8/9 was markedly upregulated; panHaLOX15 was upregulated upon ACC treatment, and panHaLOX18 was upregulated upon SA treatment. In contrast, no substantial differences in gene expression were observed following treatment with gibberellin acid (GA3), brassinosteroid (Bras), or strigolactone (Stri). Notably, methyl jasmonate (MeJA) markedly upregulated eight genes (panHaLOX3/6/10/13/14/15/19/21), all of which belong to the 13-LOX subfamily. These findings raise the possibility that the MeJA-upregulated 13-LOX genes are involved in the biosynthesis of oxylipins, including JA.

### Identification of salt stress-responsive *HaLOX* genes

The LOX gene family plays a widespread role in plant salt stress responses (Upadhyay et al., 2020; Zhang et al., 2021). However, the specific HaLOX genes involved in the salt stress response remain unidentified. Based on leaf transcriptome data collected at different time points following salt treatment, we identified candidate salt-responsive HaLOX genes using |log_2_FC| > 1 as the screening criterion. These genes included panHaLOX1, panHaLOX3, panHaLOX4, panHaLOX5, panHaLOX10, panHaLOX12, panHaLOX13, panHaLOX14, panHaLOX15, panHaLOX16, panHaLOX17, and panHaLOX20 (**Supplementary Figure 8** and **Supplementary Table 13**). Co-expression network analysis further suggested that transcription factors such as MYB, MYC, bHLH, bZIP, and ABF were highly connected to some of these candidate genes, including panHaLOX1/4/10/13/15/17 (**Figure 9A**). To further examine whether the enrichment of MYB, MYC, and ABRE related cis-elements in promoters is associated with salt-responsive transcription, we compared the enrichment of these cis-elements between salt-responsive (|log_2_FC| > 1) and non-salt-responsive HaLOX genes using a salt-stress transcriptomic dataset. We found no statistically significant differences between the two groups for any of the three cis-elements (**Supplementary Figure 9**). Nevertheless, given the complexity of promoter regulation under salt stress, the potential regulatory roles of these cis-elements in salt-triggered gene expression cannot be excluded. Given the non-significant difference in cis-element enrichment, we further characterized the expression dynamics of nine salt-induced candidate HaLOX genes.

**Figure 9:**
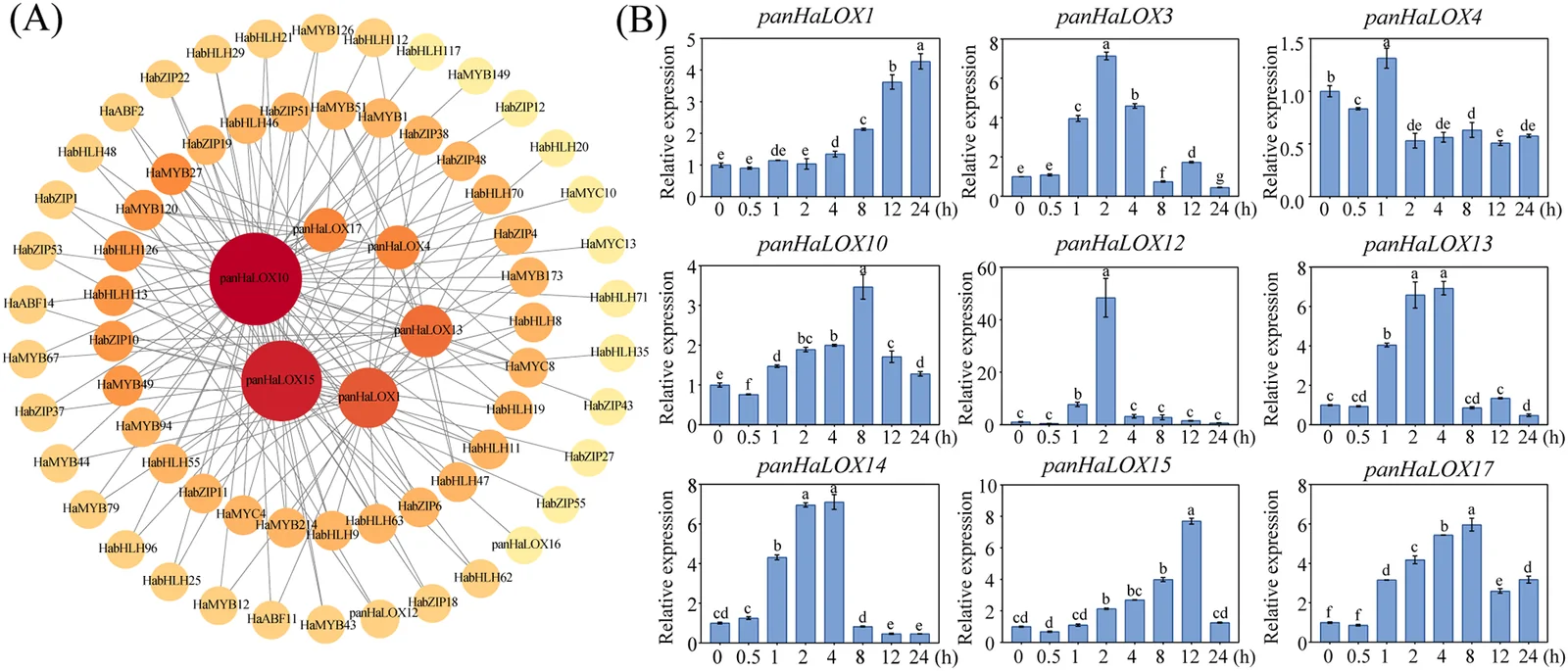
Co-expression network and expression patterns of salt stress-responsive *HaLOX* genes in sunflower leaves. **(A)** Co-expression network analysis of *HaLOX* genes and potential transcription factors under salt stress. The node size represents the degree of connectivity. The color gradient from yellow to red indicates increasing connectivity, with red nodes representing the *HaLOX* genes with the highest connectivity. **(B)** RT-qPCR validation of salt stress-responsive *HaLOX* genes in sunflower leaves. The y-axis represents the relative fold change normalized to the control (0 h). Data are shown as means ± SD of three biological replicates and normalized to *HaTubulin*. Different letters indicate significant differences (one-way ANOVA, *P* < 0.05). Different letters indicate significant differences (*P* < 0.05).

To verify the expression profiles of HaLOX genes in the tissues of sunflower plants exposed to salt stress, we selected nine genes for RT-qPCR analysis at multiple time points during exposure (0, 0.5, 1, 2, 4, 8, 12, and 24 h). Among the assessed genes, the expression of panHaLOX1 remained relatively stable during the initial stage of treatment (0–2 h), whereas a significant increase was detected at 4 h, with further rapid upregulation at 8, 12, and 24 h (**Figure 9B**). Comparatively, panHaLOX13 and panHaLOX14, which were assessed to be closely phylogenetically related, were found to be characterized by similar expression patterns under salt stress. However, despite their similar salt-stress expression patterns, the predicted numbers of cis-regulatory elements were not entirely consistent between the two genes (**Figure 7D**). These observations suggest that the similar expression patterns of panHaLOX13 and panHaLOX14 may not be solely determined by the number of predicted cis-regulatory elements. Specifically, upon an initial exposure to salt stress (0–0.5 h), the expression of both genes was stable, whereas a marked induction was observed at 1 h, with levels subsequently peaking at 2–4 h, prior to a sharp decline at 8 h, with low expression levels being maintained thereafter until 24 h. A similar, albeit more pronounced, response was observed in the case of panHaLOX4, for which a marked peak in expression occurred at 1 h, followed by a rapid decline, with subsequent maintenance at low levels from 2 to 24 h. PanHaLOX10 and panHaLOX17 were characterized by similar dynamic patterns when exposed to salt stress, with stable low expression initially being maintained between 0 and 0.5 h, followed by a gradual upregulation from 1 to 8 h and subsequent decline. In addition, although both panHaLOX3 and panHaLOX12 were rapidly induced upon exposure for 1–2 h, expression of the latter gene peaked sharply at 2 h (an approximate 48-fold upregulation), followed by a rapid return to basal levels. Comparatively, while the expression of the former also peaked at 2 h, the subsequent decline was more gradual. In contrast, we detected a significant upregulation of panHaLOX15 expression from 2 to12 h, reaching a peak at 12 h (an approximate 8-fold upregulation). Overall, the results suggest that these genes are responsive to salt stress and may be involved in the regulation of salt stress responses in sunflower.

## Discussion

Plant LOX proteins are a class of dioxygenases containing non-heme iron that play pivotal roles in plant growth and development, responses to biological and abiotic stresses, and the biosynthesis of flavor metabolites (Zhu et al., 2024). In this study, we systematically identified members of the *HaLOX* gene family members at the OGG level based on the sunflower pan-genome. This strategy enabled us to effectively overcome challenges associated with the detection of PAVs and CNVs, which have previously been difficult to identify using a single reference genome. However, although this approach can potentially provide a wealth of insights into the conservation and diversity of the *LOX* gene family, the reliability of pan-genomic analyses is vitally dependent on the completeness of genome assembly and gene annotation. To address this issue, we used the BUSCO completeness results for the 10 sunflower genomes reported by Huang et al. (2023). These results showed that 7 of these genomes were characterized by complete BUSCO proportions > 94%, whereas the levels of completeness among the remaining the remaining 3 genomes (LR1, OQP8, and HA300) were relatively lower at 85.9%, 88.1%, and 90.3%, respectively (Huang et al., 2023) (**Supplementary Table 1**). Although the incompleteness of these three genomes may have led to a slight underestimation of the true number of *HaLOX* genes, further analysis indicated that such false-negative results are unlikely to have invalidated overall conclusions. Specifically, among the dispensable OGGs (accounting for 55.6% of all OGGs), the softcore OGGs were present in the LR1, OQP8, and HA300 genomes; however, they could not be detected in several of the high-completeness genomes, including PSC8, RHA438, IR, and PI659440. With respect to shell OGGs, although the OQP8 genome was characterized by numerous gene losses, these missing genes were also absent from some of the high-completeness genomes. In addition, we found that most of the assessed genomes are deficient in the two cloud OGGs. On the basis of these distribution patterns, we infer that the main issue regarding the low-completeness genomes relates to the distinction between softcore and shell OGGs, whereas the identification and total number of both core and dispensable OGGs remain essentially unaffected. Consequently, we infer that identification of the LOX pan-gene family based on the 10 currently available sunflower genomes is feasible. Unlike the widespread PAVs, CNVs are relatively rare within the *HaLOX* family. Specifically, copy number expansion was detected in only two core OGGs, *panHaLOX1* and *panHaLOX2*, with *panHaLOX1* present in two copies in IR and *panHaLOX2* present in two copies in PI659440. This characteristic differs substantially from the widespread copy number expansion observed in other gene families, such as *APX* (Hou et al., 2026), in which core OGGs frequently undergo extensive copy number expansion. Overall, we established that PAVs within the sunflower *LOX* gene family are predominantly distributed among the dispensable OGGs, thus reflecting genetic diversity; whereas the limited number of CNVs in core OGGs may suggest constrained variation under functional purifying selection. In general, the BUSCO assessment not only confirmed the reliability of the genome assemblies used in this study, but also ensured the validity of our evolutionary analyses of PAVs and CNVs in the *HaLOX* family, thereby providing a basis for deciphering the evolutionary patterns and adaptive diversification of this family at the pan-genomic level.

Among the 27 identified *HaLOX* genes, 26 contained both the typical PLAT/LH2 domain and a complete lipoxygenase domain, whereas *panHaLOX26* lacked the PLAT/LH2 domain and retained only the complete lipoxygenase domain. Similar domain losses have also been reported for genes in *Medicago truncatula*, including *MtLOX8* and *MtLOX29* (Xu et al., 2024). Previous studies have shown that a “mini-LOX” generated by N-terminal truncation of soybean LOX1 retained catalytic activity with improved efficiency (Ivanov et al., 2010), and that an N-terminally truncated variant of melon CmLOX13 retained lipoxygenase activity in a heterologous expression system (Cao et al., 2016). These observations suggest that the catalytic core of lipoxygenases resides in the C-terminal domain and that the PLAT/LH2 domain may not be essential for catalytic activity. However, these conclusions are largely based on *in vitro* experiments or heterologous expression systems; thus, whether LOX proteins lacking the PLAT/LH2 domain are synthesized and active in planta remains to be determined. Moreover, it is also possible that *panHaLOX26*, a cloud gene detected only in the PSC8 genome, represents a truncated gene copy or pseudogene. However, phylogenetic analysis showed that *panHaLOX26* is not phylogenetically distant from the other *HaLOX* sequences, suggesting that it has not experienced relaxed selection, as is typically expected for a pseudogene. Therefore, its addition to the present sequence-based analyses should be interpreted only as a sequence-level classification based on the presence of a complete lipoxygenase catalytic domain, and should not be taken as evidence for biological function. We retained *panHaLOX26* in the family analysis and annotated its structural anomaly accordingly. Further expression profiling and enzymatic activity assays will be needed to determine whether panHaLOX26 encodes a functional protein.

The plant *LOX* gene family can be phylogenetically classified into two subfamilies, 9-LOX and 13-LOX, the numbers of each of which differ according to species. For example, in *Arabidopsis*, 9-LOX and 13-LOX contain 2 and 4 members; in rice, 6 and 8 members (Umate, 2011); in maize, 6 and 7 members (Li et al., 2025a); in wheat, 19 and 25 members (Wang et al., 2023b); in soybean,4 and 32 (Zhang et al., 2022); and in potato, 11 and 6, respectively (Zhu et al., 2024). In the present study, the 27 OGGs identified in the sunflower pan-genome contained 9 and 18 members within the 9-LOX and 13-LOX subfamilies, respectively, indicating the differential expansion of the genes in these subfamilies (**Figure 2**). Thus, it is plausible that differences among species with respect in *HaLOX* subfamily gene numbers may be linked to gene family expansion. In addition, within these two subfamilies, we observed a clustering of the 9-LOX genes *panHaLOX20*, *panHaLOX23*, and *panHaLOX25*, indicating a close evolutionary relationship. Similarly, in the 13-LOX subfamily, *panHaLOX13/14/27* clustered together, as did *panHaLOX22/24/26*, with each group, indicating a close evolutionary relationship. Likewise, in lettuce, clustering of the *LsLOX10/11/14/15* genes has been identified, and it is assumed that these multiple closely related *LOX* genes may have originated from gene duplication events.

Subfamily-level analysis of duplication modes revealed that both WGD/segmental and tandem duplications have contributed to the obesrved expansion of the *HaLOX* gene family (**Figure 4C**), albeit with pronounced differences in their relative contributions. Specifically, in the 9-LOX subfamily, in which tandem events have been the predominant mechanism of expansion (50%), we failed to identify any WGD/segmental events; whereas, the 13-LOX subfamily has mainly undergone expansion via WGD/segmental duplications (52.3%), followed by tandem duplications (27.1%). We speculate that these differences in duplication mode may reflect differences between the two sub-families with respect to evolutionary and functional divergence mechanisms. Tandem duplications generate adjacent gene clusters, with the duplicated genes often being subject to stronger selective pressure, and thus tending to diverge toward specialized functions such as plant defense (Qiao et al., 2019). This is consistent with the functional roles of the 9-LOX proteins as enzymes primarily involved in defense-related processes in plants such as insect resistance (Yuan et al., 2023). In contrast, WGD/segmental duplications contribute to increases in genome-wide copy numbers, thus giving rise to systemic redundancy and facilitating the maintenance of dosage balance in molecular signaling pathways (Freeling, 2009; Shi and Van de Peer, 2026). Correspondingly, plant 13-LOX enzymes are mainly involved in the biosynthesis of jasmonic acid and diverse stress responses (Chauvin et al., 2013; Yadav et al., 2026). Moreover, in soybean, WGD and tandem duplications are assumed to have jointly promoted LOX family expansion (Lamlom et al., 2025), whereas in rosids, 9-LOX members have smaller genetic distances than their 13-LOX counterparts (Chen et al., 2016). Overall, the 9-LOX and 13-LOX subfamilies exhibit distinct preferences for duplication modes, and this pattern may be associated with their functional divergence and divergent evolutionary trajectories.

Furthermore, transposable elements play an important role in gene duplication (Ma et al., 2023). In the present study, we found that *HaLOX* genes derived from tandem and WGD/segmental duplications were characterized by significantly higher abundance of TEs in their flanking regions compared with those generated via other modes of duplication, and that a close similarity existed between these two mechanisms and the proportions of DNA transposons and LTR retrotransposons within the resulting genes (**Figure 5B**). In addition, compared with that in the 13-LOX subfamily members, a significant enrichment of Helitron transposons was detected within the 9-LOX gene family members (**Figure 5C**). However, although it is difficult to establish a direct causal relationship between TE abundance and gene duplication, extensive evidence has indicated that transposon-mediated gene capture is an important mechanism for generating new genes and gene duplications in plant genomes (Cerda-Hernandez and Bureau, 2026). Accordingly, we speculate that an enrichment of TEs may have contributed to the expansion of the *HaLOX* gene family, although this hypothesis remains to be further validated based on precise identification of TE insertion sites and their collinearity with duplication events.

Analysis of selection pressures acting on the *HaLOX* gene familysuggested a relatively conserved evolutionary trajectory (**Figure 6**). *K*a/*K*s analysis showed that a majority of the homologous gene pairs had *K*a/*K*s ratios < 1, indicating that they have been subject to purifying selection and that *HaLOX* genes are highly conserved among sunflower accessions. However, for certain homologous gene pairs, *K*a/*K*s-based screening detected signals of positive selection signals in three 13-LOX genes (*panHaLOX10*, *panHaLOX14*, and *panHaLOX17*), suggesting that some 13-LOX paralogs may have been subjected to a relaxation of evolutionary constraints, potentially allowing the accumulation of non-synonymous mutations. To further evaluate the statistical support for this signals, we performed likelihood-based codon substitution analyses, among which, BUSTED analysis supported the occurrence of significant episodic positive selection in *panHaLOX10* and *panHaLOX17*, but not in *panHaLOX14*. Thus, rather than indicating adaptive evolution, the initial *K*a/*K*s signal for *panHaLOX14* may reflect relaxed purifying selection. This distinction highlights the value of likelihood-based methods for distinguishing episodic positive/diversifying selection from constraint relaxation, but they do not demonstrate adaptive phenotypic effects of specific substitutions.

With respect to LOX proteins activity, the PLAT/LH2 domain has been established to mediate membrane anchoring and substrate delivery, and mutations in this domain have been linked to reduced catalytic efficiency (Joshi et al., 2013). For example, CRISPR/Cas9-induced mutations in the PLAT/LH2 domain of soybean Lox-2 have been demonstrated to reduce enzyme activity by up to 25.49%, thereby confirming that alterations in this region can directly influence catalytic performance (Patel et al., 2025). We thus speculate that the sites inferred to be under episodic positive selection within the PLAT/LH2 domain of panHaLOX10 (Thr111→Leu) and panHaLOX17 (Ser20→His) might affect enzyme activity, although this remains to be tested. Given that these substitutions alter residue properties (i.e., Thr→Leu involves replacement of a polar residue with a hydrophobic residue, whereas Ser→His introduces a charged residue), they might influence membrane anchoring or substrate accessibility. Furthermore, the C-terminal residues of these enzymes serve as key ligands for catalytic iron, thus, mutations in this region can have a direct and potentially pronounced effect on enzymatic activity (Okamoto et al., 2005). For example, the Val→Gln substitution detected at site 899 of panHaLOX17 in the present study lies near the C terminus and results in the replacement of a hydrophobic residue with a polar uncharged glutamine. Collectively, these sites, which were inferred to be under episodic positively selection and are located within the PLAT/LH2 domain and near the C terminus, might affect membrane association, substrate accessibility, or catalytic efficiency, potentially contributing to functional divergence among sunflower 13-LOX paralogs. However, direct functional evidence is currently lacking, and these inferences warrant further experimental validation.

Core genes are evolutionarily highly conserved essential genes that typically maintain elevated basal transcription levels in most tissues, as exemplified by barley *bHLH* core genes (Tong et al., 2025), which are closely associated with their conserved biological functions. Among the 12 *HaLOX* core genes identified in this study, half (*panHaLOX1*/*2*/*3*/*9*/*10*/*11*) were highly expressed in at least five sunflower tissues, whereas the remaining six were preferentially expressed in only a few specific tissues. Notably, *panHaLOX1* and *panHaLOX9* are the only two pollen-specific genes with high expression levels in this family (**Figure 8A**). JA plays a pivotal role in regulating pollen maturation, filament elongation, and anther dehiscence, and its biosynthesis is dependent on the catalytic activity of 13-LOX (Ghorbel et al., 2021). In rice, tight regulation of JA biosynthesis in anthers induces male sterility (Fang et al., 2024). Similarly, in *Arabidopsis*, 13-LOX members (*AtLOX3* and *AtLOX4*) participate in male fertility regulation, and exogenous JA treatment effectively restores fertility (Caldelari et al., 2011). *PanHaLOX1* is the only member of the 13-LOX subfamily with pollen-specific expression. Considering that *panHaLOX1* is the only pollen-specific member of the 13-LOX subfamily with pollen-specific expression (**Figure 8A**), the study hypothesized that it may participate in JA biosynthesis and fertility regulation in sunflower anthers. However, the molecular mechanisms require further experimental validation. In addition, we found that *panHaLOX12* exhibited extremely low expression across all normal sunflower tissues. However, its promoter region is enriched with a relatively large number of stress- and phytohormone-responsive cis-elements, such as MYB, G-box, and ABRE (Wang et al., 2021; Fiallos-Salguero et al., 2023; Lei et al., 2024), and it is strongly induced by salt stress (**Figure 9B**). These results suggest that this gene may be a salt stress-specific response gene. Overall, the divergent expression patterns among core genes suggest potential functional differentiation and are related to their possible distinct regulatory roles in plant growth, development, and stress responses.

Unlike the core genes, some softcore and shell genes exhibit low expression levels and tissue-specific expression patterns (Tong et al., 2025). In this study, six softcore genes of the *HaLOX* family displayed distinct expression divergence. Only the 9-LOX subfamily member *panHaLOX18* showed root-specific expression, whereas the remaining five 13-LOX members (*panHaLOX13*/*14*/*15*/*16*/*17*) maintained high expression levels across multiple tissues (**Figure 8A**). These results suggest that softcore genes may functionally compensate for the insufficient tissue expression of core genes through expression dosage. Evolutionary and expression analyses revealed that *panHaLOX16* and *panHaLOX17*, which are evolutionarily close paralogs, exhibited highly consistent tissue expression patterns, thereby suggesting potential functional redundancy in their roles in developmental regulation. However, their cis-element distributions were markedly different (**Figure 7D**). This discrepancy may be partly attributable to the inherent limitations of cis-element prediction and the complexity of promoter regulation. In particular, the *panHaLOX17* promoter was enriched with MYB, MYC, G-box, and ABRE stress- and phytohormone-responsive elements (Wang et al., 2021; Fiallos-Salguero et al., 2023; Lei et al., 2024; Bao et al., 2025). Based on the co-expression network and RT-qPCR validation (**Figure 9**), *panHaLOX17* likely exhibits a complex transcriptional pattern and is a candidate gene potentially involved in the salt stress response. Furthermore, both the softcore gene *panHaLOX15* and the shell genes *panHaLOX19*/*21* were greatly induced by MeJA and highly expressed in the roots (**Figure 8B**). This suggests that these softcore and shell genes may participate in regulating the root JA signaling pathway and play a crucial role in JA synthesis and phytohormonal responses in sunflower roots (Sun et al., 2022). Although some softcore genes may be dispensable in this study, it remains possible that one or more of them are not dispensable. Overall, core genes established the genetic foundation of sunflower germplasms, whereas softcore and shell genes enhanced genetic variation and functional diversity among different germplasms through expression divergence and pathway-specific regulation. This ultimately contributed to the genetic diversity and functional plasticity of the sunflower *HaLOX* family.

The identification of *LOX* genes associated with salt stress regulation has crucial implications for breeding salt-tolerant crops (Yu et al., 2019; Upadhyay et al., 2020). The JA signaling pathway positively regulates plant salt tolerance, and 13-LOX subfamily genes encode key initiating enzymes in JA biosynthesis (Li et al., 2025b). In this study, cis-element analysis revealed that stress- and phytohormone-responsive elements were predominant among the cis-elements predicted in *HaLOX* promoters, with MYB, MYC, G-box, and ABRE being the most frequently detected (**Figure 7D**). However, the enrichment of promoter elements was based on prediction and requires experimental validation. Moreover, no significant differences in MYB, MYC, and ABRE element enrichment were observed between salt-responsive and non-salt-responsive *HaLOX* genes (**Supplementary Figure 9**). Nonetheless, these enriched elements may still have potential regulatory roles in salt stress regulation and remain to be functionally validated. Furthermore, the co-expression network suggested potential regulatory relationships between the salt stress-responsive 13-LOX genes and transcription factors (MYB, bHLH, bZIP, MYC, and ABF) (**Figure 9A**). This suggests that these transcription factors may contribute to the transcriptional regulation of *HaLOX* genes under salt stress via the corresponding cis-elements.Temporal expression profiling of the nine 13-LOX genes under salt stress conditions using RT-qPCR confirmed distinct dynamic expression patterns (**Figure 9B**). Notably, *panHaLOX3/10/13/14/15* were also upregulated by MeJA (**Figure 8B**). Given the enrichment of JA signaling-related elements (e.g., MYC) in the promoter regions of these 13-LOX genes, it is tempting to speculate that MeJA induction and salt stress responses may not be entirely independent biological processes, suggesting that these genes may participate in salt stress responses through JA-related transcriptional regulation. A similar regulatory mechanism has been reported in soybean, where *GmLOX6* regulates salt tolerance by initiating JA biosynthesis (Li et al., 2025b). However, whether these candidate genes regulate salt tolerance in sunflower through a similar oxylipin-related mechanism, potentially involving JA, remains to be further validated. In addition, although *panHaLOX10* and *panHaLOX17* are salt-stress-responsive genes and have been undergo substantial positive selection, the current evidence is insufficient to establish a direct causal relationship between putatively positively selected amino acid substitutions and salt tolerance. Therefore, future work should functionally validate the regulatory roles of these candidate genes in response to salt stress and elucidate the association between amino acid variations and adaptive evolution to salt stress.

## Data Availability

The datasets presented in this study can be found in online repositories. The names of the repository/repositories and accession number(s) can be found in the article/**Supplementary Material**.
