# Supplementary material for: Pan-genome analysis of the sunflower LOX gene family: evolution, expression, and salt stress-responsive candidate genes
Source: Front Plant Sci. 2026 Oct 7;17:1940299. doi: 10.3389/fpls.2026.1940299 (PMC13684364; doi:10.3389/fpls.2026.1940299)
Supplement: Supplementary file 1 [file DataSheet1.pdf]

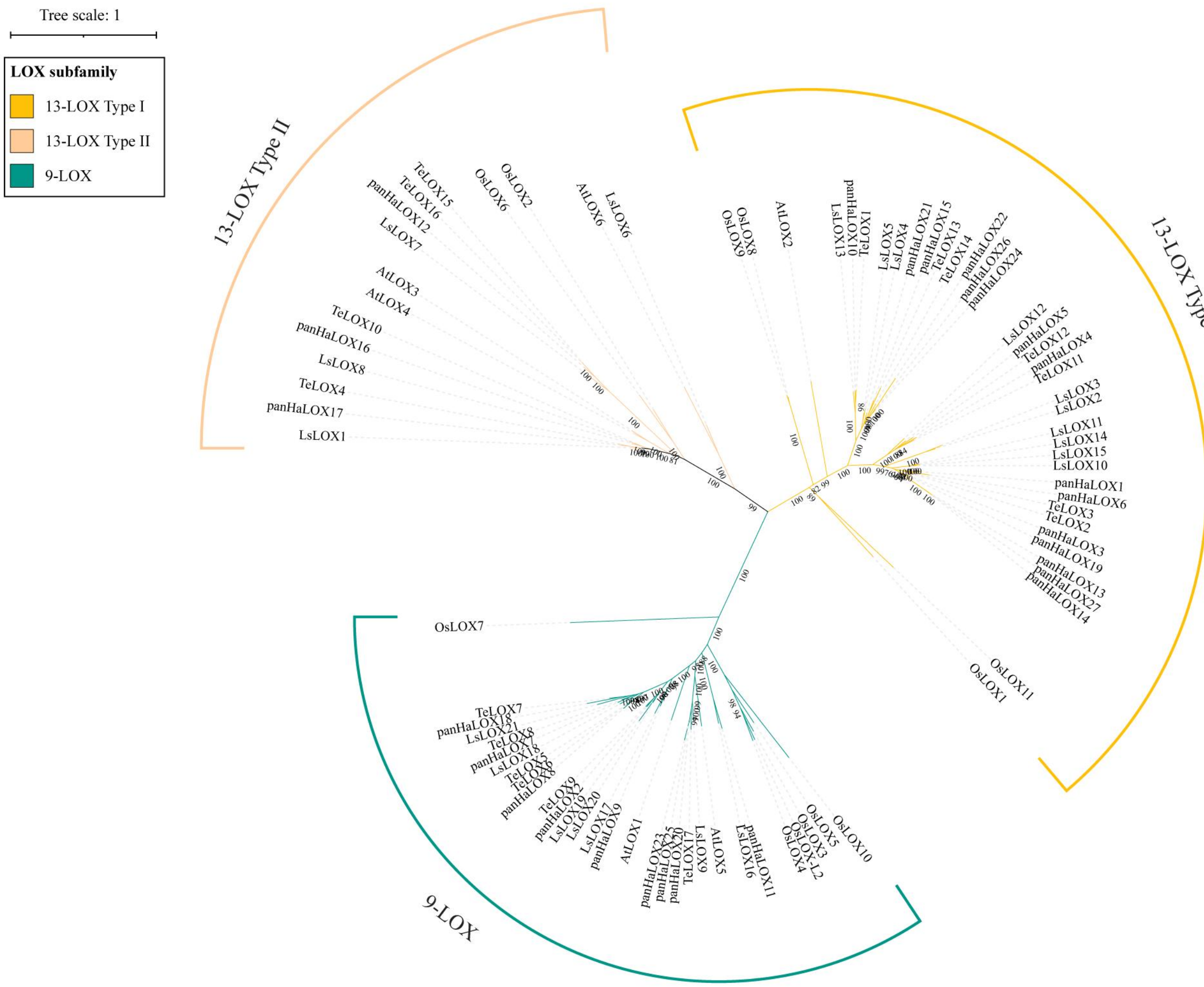


**Supplementary Figure 1.** Phylogenetic classification of LOX proteins from rice, marigold, lettuce, *Arabidopsis,* and sunflower.

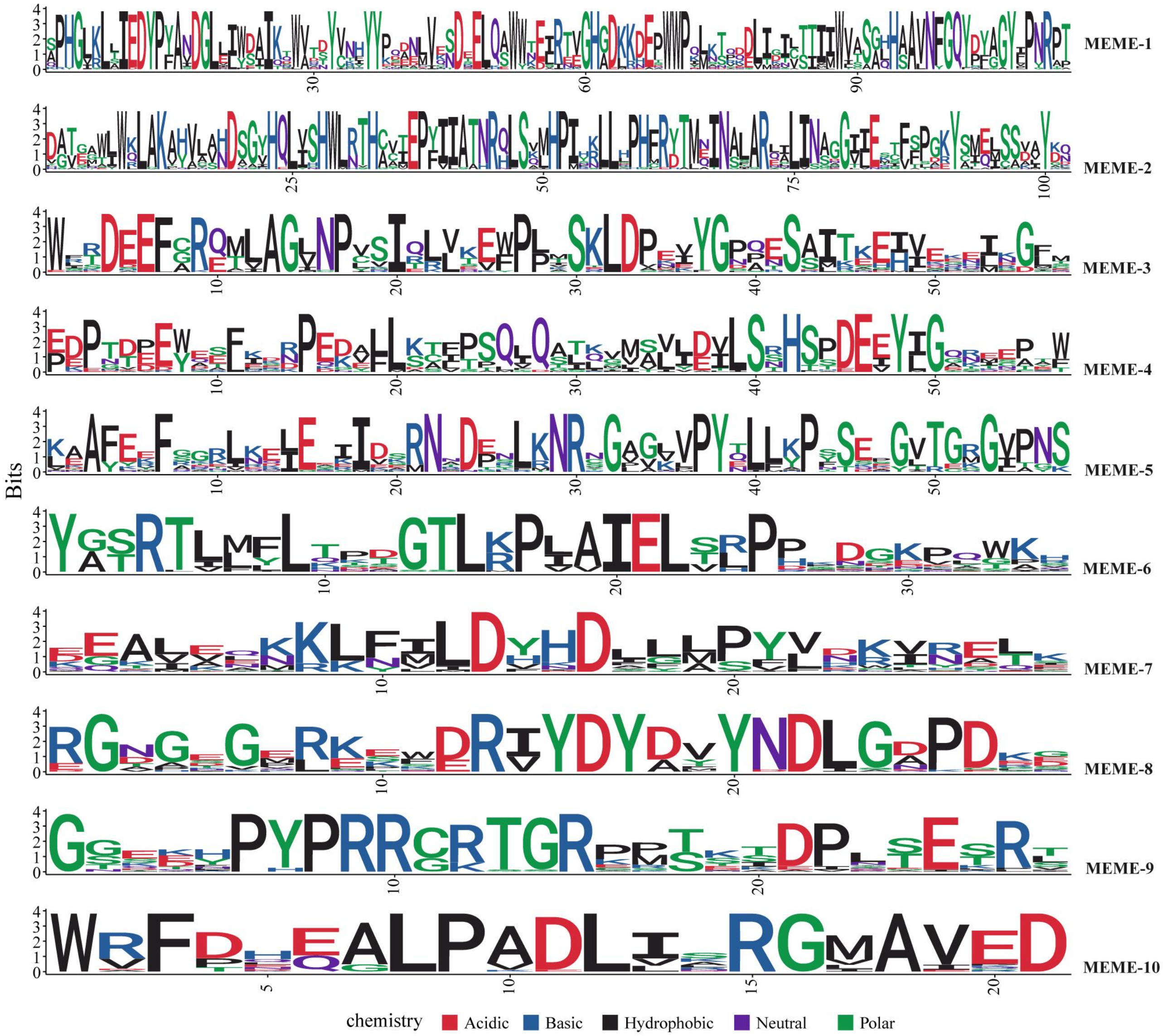


**Supplementary Figure 2.** Amino acid profiles of 10 MEME motifs identified in 27 sunflower *HaLOX* orthologous gene groups (OGGs).

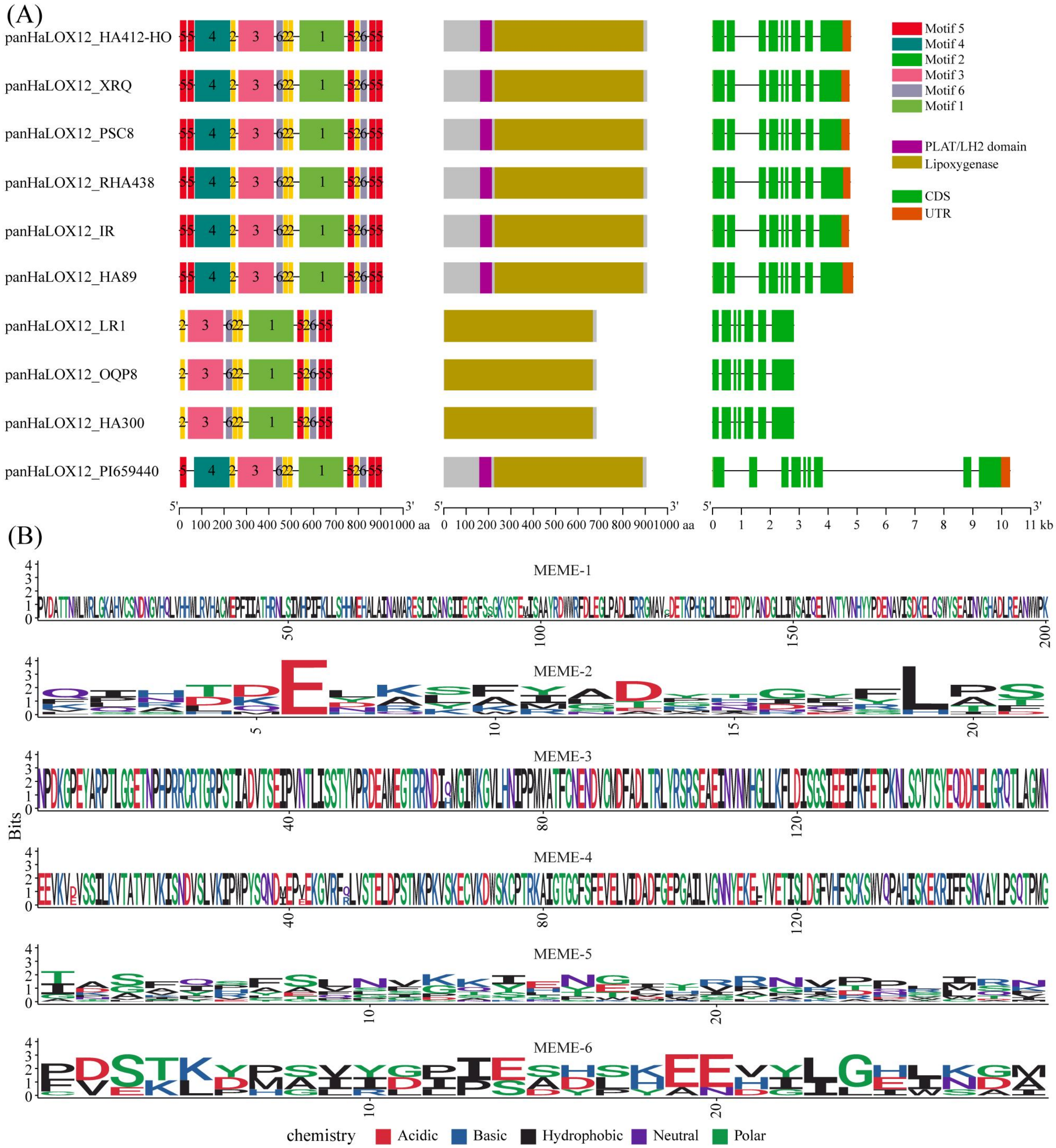


**Supplementary Figure 3.** Characterization of conserved motifs, protein domains, and gene structures of *panHaLOX12* across 10 sunflower accessions. (A) Conserved motifs, protein domains, and gene structures arranged from left to right. (B) Sequence logos of the six conserved motifs detected in panHaLOX12 proteins.

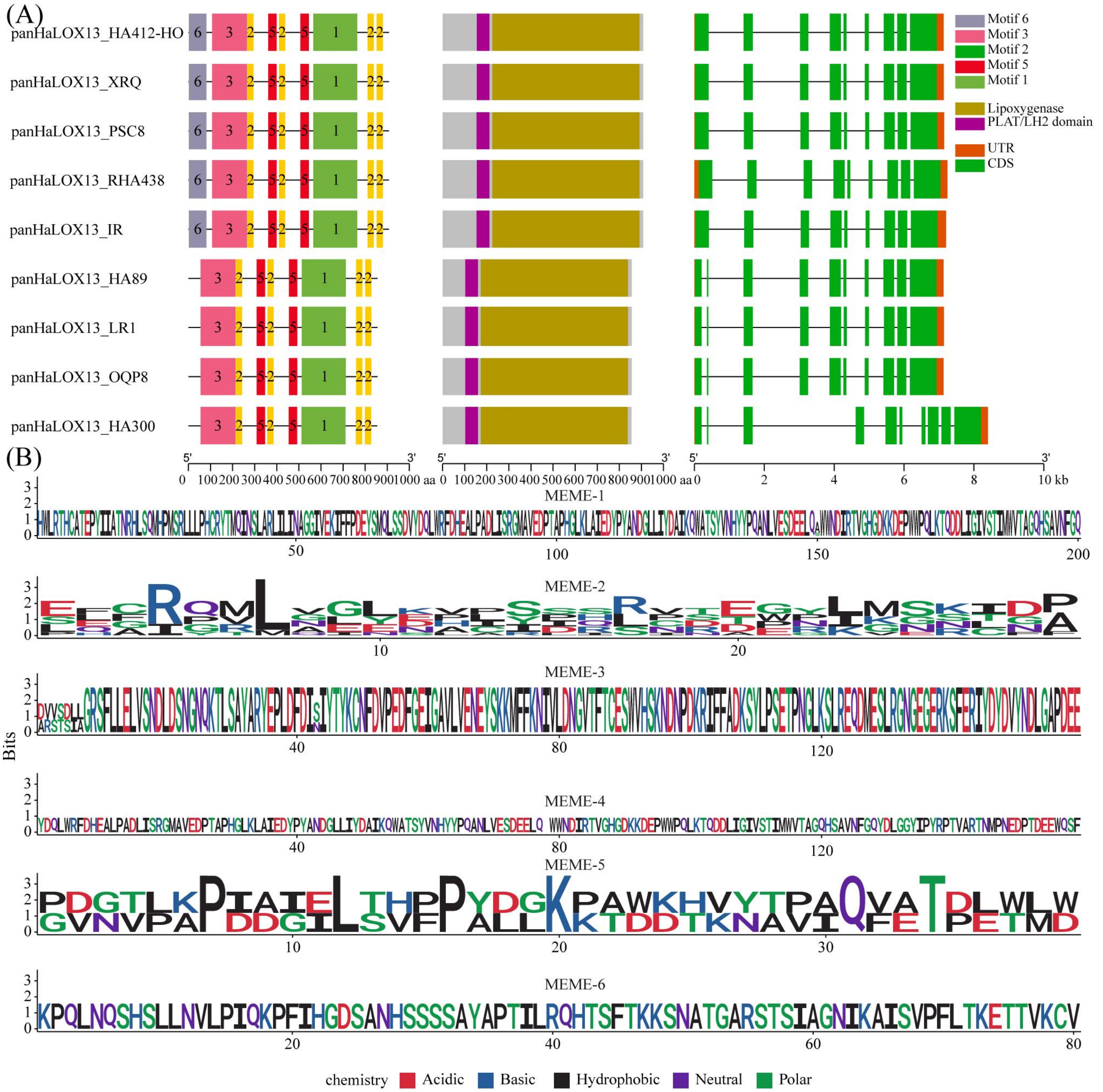


**Supplementary Figure 4.** Characterization of conserved motifs, protein domains, and gene structures of *panHaLOX13* across nine sunflower accessions. (A) Conserved motifs, protein domains and gene structures arranged from left to right. (B) Logos for six conserved motifs detected in panHaLOX13 proteins.

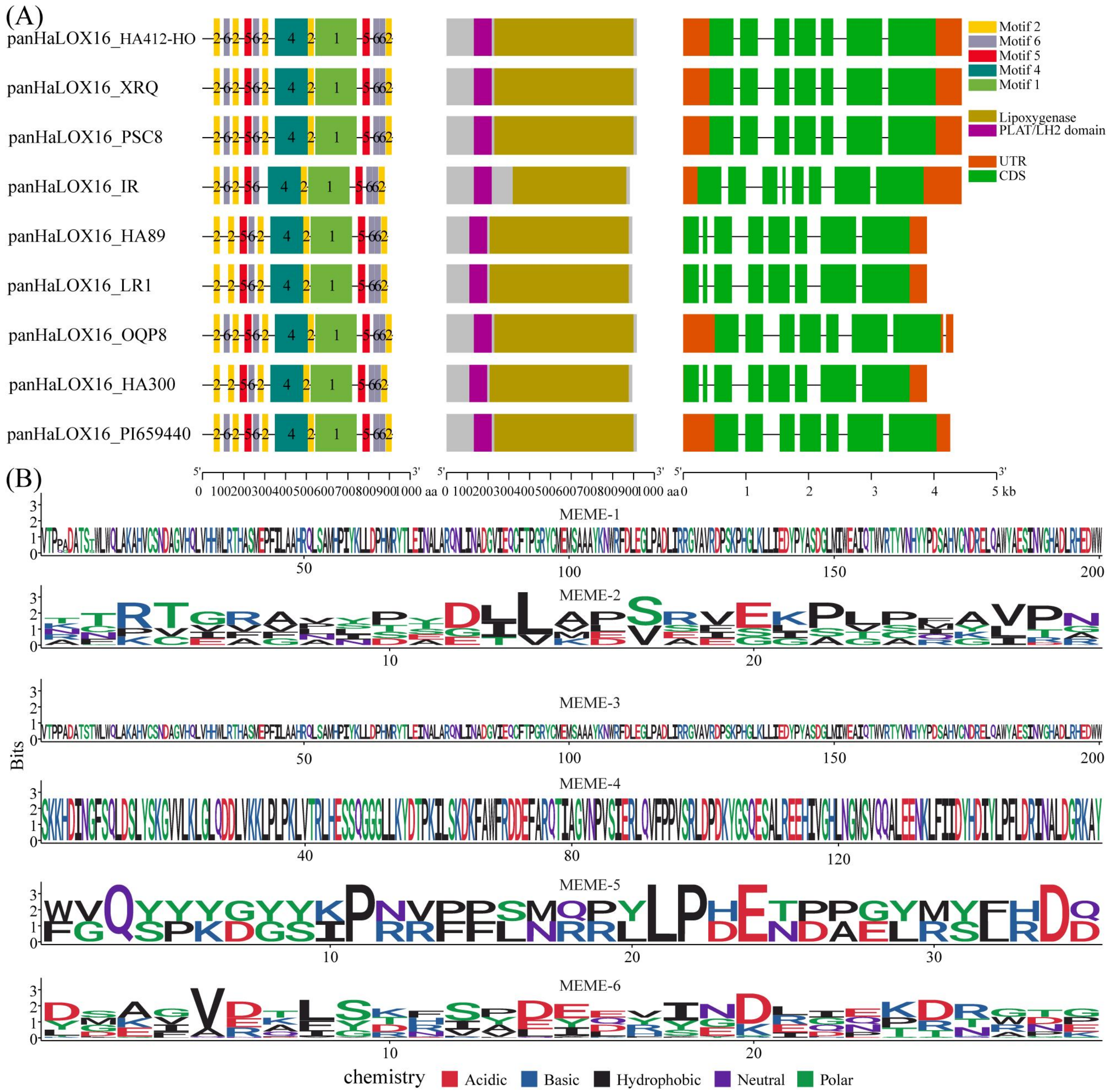


**Supplementary Figure 5.** Characterization of conserved motifs, protein domains, and gene structures of *panHaLOX16* across nine sunflower accessions. (A) Conserved motifs, protein domains, and gene structures arranged from left to right. (B) Sequence logos of six conserved motifs identified in panHaLOX16 proteins.

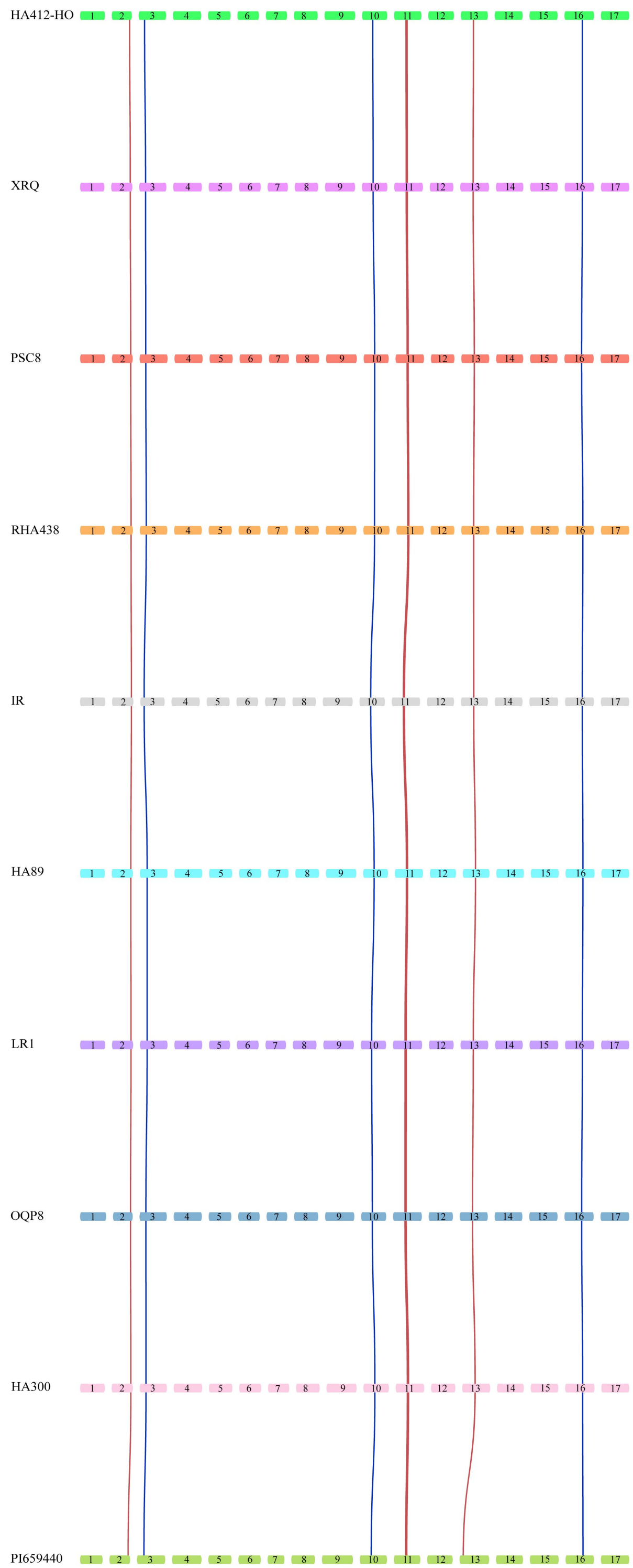


**Supplementary Figure 6.** Syntenic relationships of *HaLOX* genes across the 10 sunflower accessions.

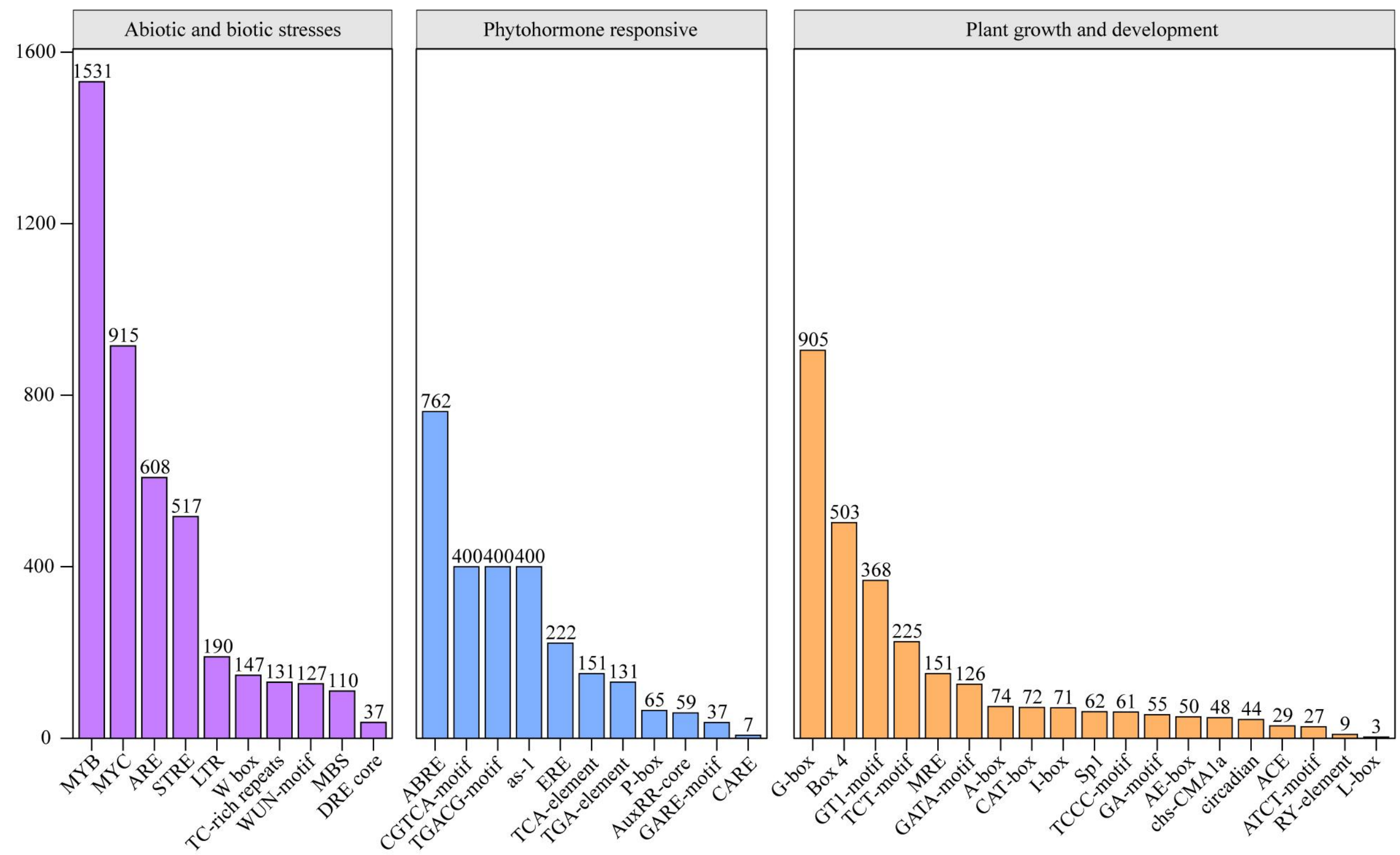


**Supplementary Figure 7.** Cis-regulatory element numbers in *HaLOX* promoters across the sunflower pan-genome.

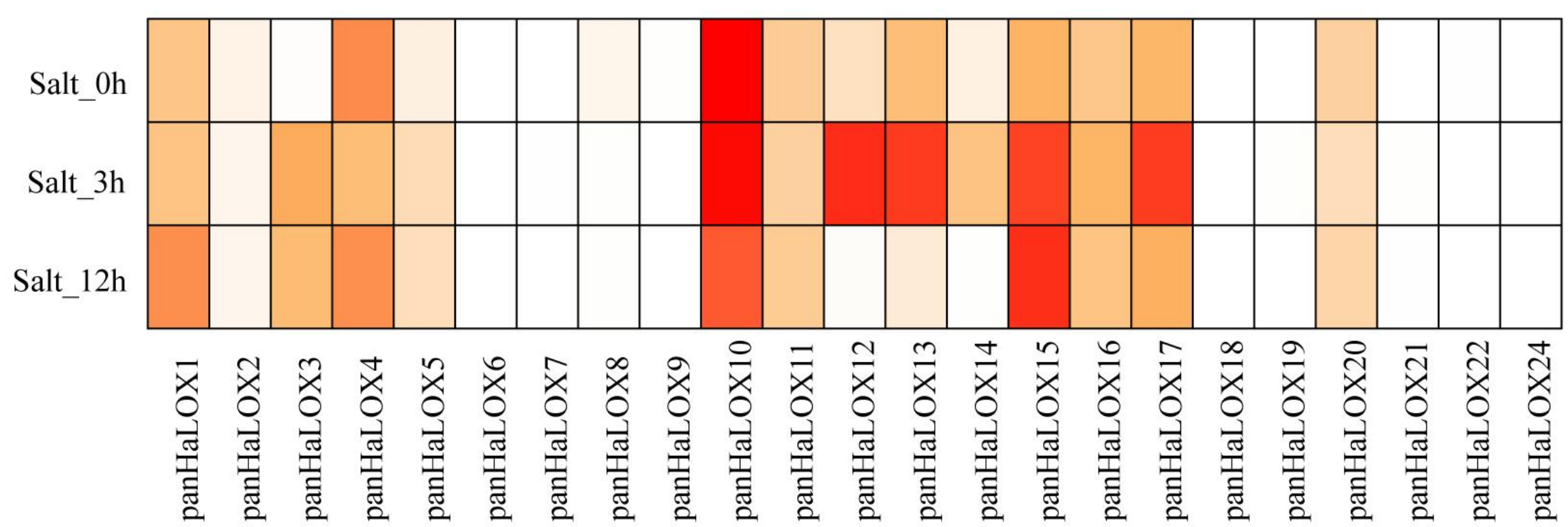


**Supplementary Figure 8.** Heatmap showing the expression patterns of *HaLOX* genes in sunflower leaves under salt stress at 0 h, 3 h, and 12 h. Gene expression values were normalized using $log_2$(TPM+1) transformation.

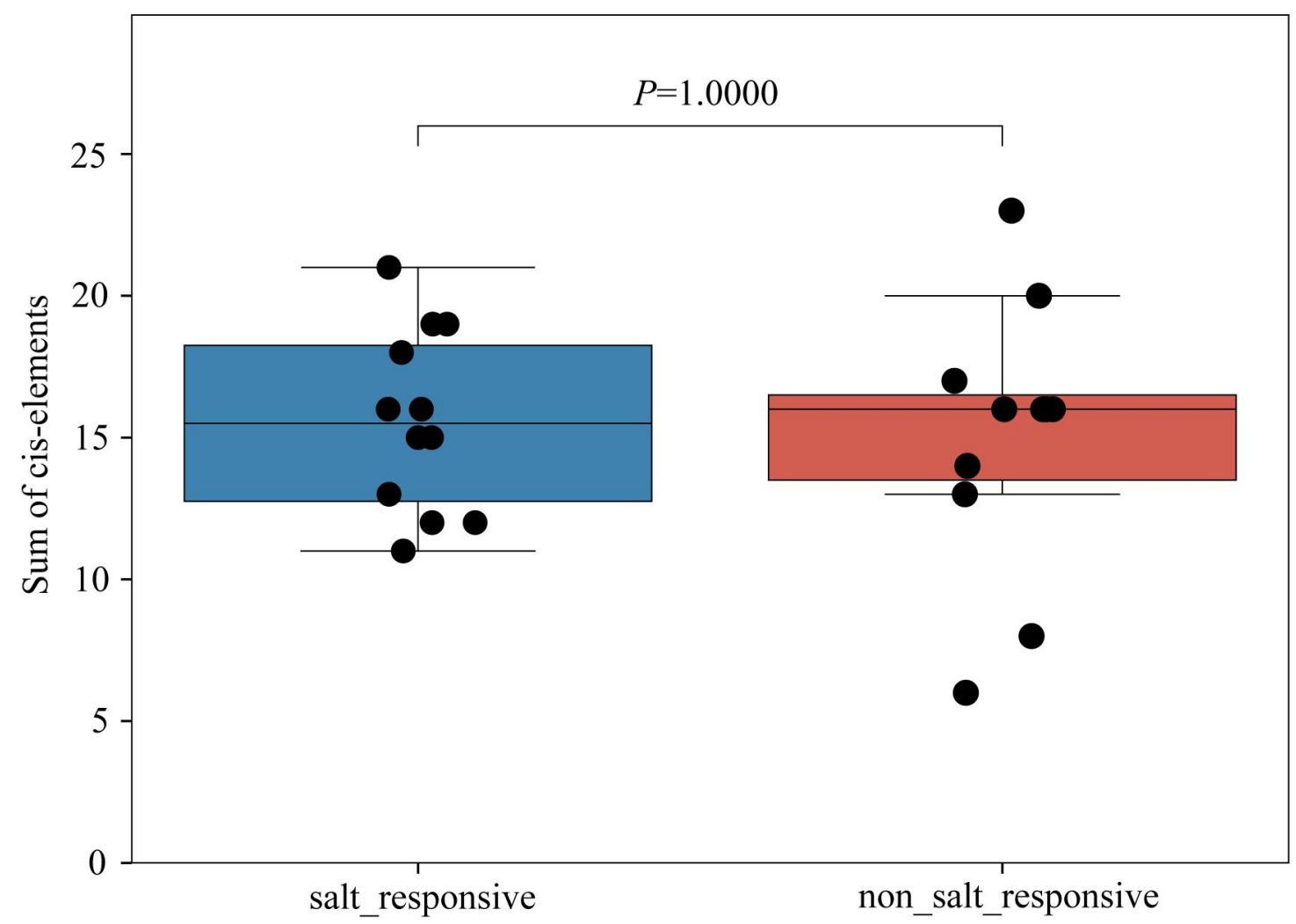


**Supplementary Figure 9.** Comparison of MYB, MYC, and ABRE cis-element numbers between salt-responsive and non-salt-responsive *HaLOX* genes.
